# HOXC9 Regulates Formation of Parachordal Lymphangioplasts and the Thoracic Duct in Zebrafish via Stabilin 2

**DOI:** 10.1371/journal.pone.0058311

**Published:** 2013-03-06

**Authors:** Sandra J. Stoll, Susanne Bartsch, Jens Kroll

**Affiliations:** 1 Department of Vascular Biology and Tumor Angiogenesis, Center for Biomedicine and Medical Technology Mannheim (CBTM), Medical Faculty Mannheim, Heidelberg University, Mannheim, Germany; 2 Division of Vascular Oncology and Metastasis, German Cancer Research Center (DKFZ-ZMBH Alliance), Heidelberg, Germany; Feinberg Cardiovascular Research Institute, Northwestern University, United States of America

## Abstract

HOXC9 belongs to the family of homeobox transcription factors, which are regulators of body patterning and development. HOXC9 acts as a negative regulator on blood endothelial cells but its function on lymphatic vessel development has not been studied. The hyaluronan receptor homologs stabilin 1 and stabilin 2 are expressed in endothelial cells but their role in vascular development is poorly understood. This study was aimed at investigating the function of HOXC9, stabilin 2 and stabilin 1 in lymphatic vessel development in zebrafish and in endothelial cells. Morpholino-based expression silencing of HOXC9 repressed parachordal lymphangioblast assembly and thoracic duct formation in zebrafish. HOXC9 positively regulated stabilin 2 expression in zebrafish and in HUVECs and expression silencing of stabilin 2 phenocopied the HOXC9 morphant vascular phenotype. This effect could be compensated by HOXC9 mRNA injection in stabilin 2 morphant zebrafish embryos. Stabilin 1 also regulated parachordal lymphangioblast and thoracic duct formation in zebrafish but acts independently of HOXC9. On a cellular level stabilin 1 and stabilin 2 regulated endothelial cell migration and in-gel sprouting angiogenesis in endothelial cells. HOXC9 was identified as novel transcriptional regulator of parachordal lymphangioblast assembly and thoracic duct formation in zebrafish that acts via stabilin 2. Stabilin 1, which acts independently of HOXC9, has a similar function in zebrafish and both receptors control important cellular processes in endothelial cells.

## Introduction

The complex bodies of vertebrates require capillary networks to distribute gases, nutrients, cells and molecules through the body. Blood vessels perform these tasks but their function is incomplete without a functional lymphatic network [1], [2]. The lymphatic system consists of endothelium-lined, blind-ended capillaries, which reabsorb the extravasated fluids -containing macromolecules and cells- from the capillary bed and return it to the blood vessels via the thoracic duct [2], [3]. Angiogenesis, the formation of blood vessels from pre-existing ones, is a major focus of researchers since decades whereas lymphangiogenesis is less well understood. Yet, it is important to understand its mechanisms because lymphatic vessels are involved in several pathological processes such as inflammation, wound healing and tumor metastasis [2]. Lymphatic endothelial cells (LECs) originate from the cardinal vein from a subpopulation of endothelial cells expressing VEGFR-3 and Lyve-1. In these endothelial cells transcription factors SOX18 and Prox1 are induced and provoke LEC differentiation. Subsequently, LECs sprout laterally and form the primary lymph sac from which the lymphatic system evolves via endothelial sprouting [1], [2].

Homeobox (HOX) genes constitute a family of transcription factors characterized by a 61-amino acid long homeodomain. They regulate embryonic development and anterior-posterior body pattern formation. Furthermore they are important for physiological and pathophysiological processes in the adult [4]. Several HOX genes have been identified as regulators of angiogenic processes such as migration, tube formation, vessel maturation and angiogenic activation in tumor angiogenesis [4], [5]. However, most HOX target genes and their underlying mechanisms are unknown. We have recently identified HOXC9 as a regulator of endothelial cell quiescence keeping endothelial cells in a resting state, which is achieved by inhibition of the proangiogenic cytokine interleukin 8 (IL-8), a direct target of HOXC9 [6], [7]. HOXC9 is expressed in blood vessels in mice [8] and zebrafish, where it is highly present in the cardinal vein [6].

The transmembrane proteins stabilin 1 and stabilin 2 belong to the family of fasciclin-like hyaluronan receptor homologues. They consist of seven fasciclin-like adhesion domains, 18–20 epidermal growth-factor domains, one X-link domain and three to six hyaluronan-binding motifs [9]. They are expressed in sinusoidal endothelial cells in the liver, spleen and lymph nodes and stabilin 1 is additionally expressed in alternatively activated macrophages [9]. Stabilin 1 and 2 act as scavenger receptors which clear the blood of waste-products. Double knockout of stabilin 1 and stabilin 2 in mice leads to nephropathy due to impaired hepatic clearance; yet a lymphatic vessel phenotype has not been reported in these mice [10]. Stabilin 1 has been shown to have angiogenesis-modulating functions in the tube-formation assay [11]. It is expressed in lymphatic vessels where it mediates lymphocyte migration through the vascular and lymphatic endothelium [12], [13]. Stabilin 1 also regulates cell-cell adhesion and thus influences pathological processes such as inflammation and metastasis [14]. Stabilin 2 interacts with the integrin αMβ2 and mediates lymphocyte adhesion to the liver sinusoidal endothelium [15]. Under pathological conditions, stabilin 2 is detected in macrophages, endothelial cells and smooth muscle cells in atherosclerotic plaques [16]. Interestingly, stabilin 2 knockout mice showed not only elevated hyaluronan levels but also decreased tumor metastasis by inhibiting the rolling and tethering of B16 cells to lung endothelial cells [17].

Expression data in zebrafish have identified HOXC9 expression in the cardinal vein whereas stabilin 2 is expressed in the head and trunk vasculature and also strongly in the cardinal vein [6], [18]. As zebrafish lymphatic vessels originate in the cardinal vein, this is a first indication for a potential involvement of HOXC9 and stabilin 2 in lymphatic development [19], [20]. Zebrafish have become a commonly used model for studying lymphangiogenesis during recent years. Using lymphatic markers and advanced imaging techniques it was demonstrated that the zebrafish lymphatic system shares many morphological, functional and molecular similarities with other vertebrates [3] ^,^ [21]. Further advantages of zebrafish like optical clarity, high number of eggs, availability of transgenic lines and easy genetic manipulation highlights the zebrafish as an attractive model for research in vascular biology [22].

In this study we demonstrate, that HOXC9 regulates stabilin 2 expression in zebrafish and in cultured endothelial cells. Expression silencing of HOXC9 and stabilin 2, but also of stabilin 1, regulates lymphatic development in zebrafish and important endothelial functions *in vitro*. Thus the data identified a novel function for HOXC9, stabilin 1 and stabilin 2 in regulating vascular development in zebrafish.

## Materials and Methods

### Ethics Statement

Isolation of HUVECs was approved by the ethics committee (Medizinische Ethik-Kommission II of the Medical Faculty Mannheim, Heidelberg University, approval no. 2007-31N-MA). The approval includes the consent of the donators of the umbilical cords. Consent was given verbally after the women were provided with handouts (patient information) explaining the purpose of the study. This procedure was approved by the ethics committee.

### Zebrafish Lines, Cell Lines, Antibodies and Reagents

Embryos of AB wildtype and the *tg(fli1:EGFP)* line [23] were raised and staged as described [24]. Embryos were kept in E3 solution at 28.5°C with or without 0.003% 1-phenyl-2-thiourea (Sigma) to suppress pigmentation and staged according to hours postfertilization (hpf) [25]. Human umbilical vein ECs (HUVECs) were freshly isolated from human umbilical veins of newborns by collagenase digestion [26] and cultured in endothelial cell growth medium EGM-2 (PromoCell) supplemented with 10% heat-inactivated fetal calf serum and antibiotics. We used following antibodies for this study: mouse anti-HOXC9 (ab50839) (Abcam), rabbit anti-stabilin-1 (AB6021) (Millipore), goat anti-Actin (I-19) (Santa Cruz Biotechnology), rabbit anti-GFP (A-11122) (Invitrogen) and HRP-conjugated antibodies (DAKO). Vascular endothelial growth factor (VEGF) was purchased from R&D Systems (VEGF-A) and PeproTech (VEGF-C).

### Transfection and Viral Transduction of HUVECs

Small interfering RNAs for HOXC9 (ID3678), Stab1 (siRNA1: IDs31005, siRNA2: IDs31006), Stab2 (siRNA1: IDs23178, siRNA2: ID136773) and a validated non-targeting negative control siRNA were synthesized by Ambion. HUVECs were transfected with the indicated siRNA (final concentration: 200 nM) using Oligofectamine (Invitrogen). The transfection medium was replaced after 4 h by ECGM containing 10% FCS and the cells were incubated for another 48 h. Adenoviruses were produced according to the ViraPower Adenovirus Expression Systems protocol (Invitrogen). The full length human HOXC9 or Cherry sequences were cloned into the adenovector and for the transduction a multiplicity of infection (MOI) of 35 was used.

### Western Blot Analysis

Cells were washed with PBS and lysed in buffer (150 mM NaCl, 50 mM Tris-HCl, pH 7.4, 1% NP40, 10 mM EDTA, 10% glycerol, and protease inhibitors). Zebrafish embryos were deyolked, lysed in the same buffer, followed by homogenization with a syringe. Cells and zebrafish lysates were incubated 30 min on ice with agitation. The protein lysates were boiled in Laemmli buffer, separated by SDS-PAGE, transferred to nitrocellulose membrane and incubated with the indicated antibodies, followed by incubation with Western blot detection reagent (Perbio Science). Western blot signals were quantified using Gel-Pro Analyzer 6.0, INTAS and normalized to its respective loading controls.

### In vitro Angiogenesis Assay and Apoptosis Assay

HUVECs were transfected with siRNA and incubated for 48 h before the *in vitro* assays were performed. Endothelial migration and sprouting was done as described [6], [27]. To investigate on cell apoptosis, caspase 3/7 activity was measured using the Caspase-Glo® 3/7 Assay System (Promega) according to the manufacturer’s protocol. As a positive control cells were stimulated with 250 nM staurosporin for 2 h at 37°C.

### RT-PCR

Total RNA was isolated from zebrafish embryos or HUVECs using the RNeasy Mini-Kit (Qiagen) following the manufacturer’s protocol. First-strand cDNA was generated from normalized RNA amounts using random hexamer primers and the Superscript II kit (Invitrogen). RT-PCR was performed with specific primer pairs: zebrafish actin (413 bp fragment, forward: CTTGCGGTATCCACGAGAC, reverse: GCGCCATACAGAGCAGAA; Program: 94°C for 3 min (94°C for 45 s, 55°C for 45 s, 72°C for 1 min)×35, 72°C for 10 min), zebrafish stab2 (658 bp fragment, forward: CTGTGTAGCAGCGGTTTGAA, reverse: ACAGTGTGATGGCACTGGAG; Program: 94°C for 3 min (94°C for 45 s, 54°C for 1 min, 72°C for 1 min)×30, 72°C for 10 min), zebrafish stab1 (550 bp fragment, forward: AATGTTTGGATGGCTTGGAG, reverse: TAGCATGTGCAATTGCGTTC; Program: 94°C for 3 min (94°C for 45 s, 56°C for 1 min, 72°C for 1 min)×33, 72°C for 10 min), human stab1 (480 bp fragment, forward: AGGACTGCCGCTACGAAGTA, reverse: CACTGCCCTGCTGTGTGTAG; Program: 94°C for 3 min (94°C for 45 s, 56°C for 1 min, 72°C for 1 min)×33, 72°C for 10 min), human stab2 (237 bp fragment, forward: TCTGAAGGCAGGTCTCACCTA, reverse: CTGGGGAGCAGAAATTTTGTA; Program: 94°C for 3 min (94°C for 45 s, 53°C for 1 min, 72°C for 1 min)×33, 72°C for 10 min), human TBP (forward: GGA TCA GAA CAA CAG CCT GCC, reverse: CCT GTG TTG CCT GCT GGG ACG; Program: 94°C for 3 min (94°C for 45 s, 51°C for 1 min, 72°C for 1 min)×33, 72°C for 10 min).

### Injections of Morpholinos and mRNA

Morpholinos were diluted in 0,1M KCl to concentrations of 2–12 µg/µl. One nanoliter of each dilution was injected through the chorion of 1-cell or 2-cell stage embryos. The following translational/splicing-blocking (TB/SB) antisense morpholinos (Gene Tools) were used:

### Stab2-ATG-Mo

5′-CTAGAAGGAACGGCATGATGATTAC-3′ (9 bases 5′ of ATG)

*Stab2-Ex2-Mo:*

5′-CGCTTCTGAAAACAGACACAATCAC-3′ (targeting intron1-exon2 junction)

*Stab2-Ex9-Mo:*

5′-GAGCCTGTGGAAAACAGCAAACTGT-3′ (targeting intron8-exon9 junction)

*Stab1-Ex3-Mo:*

5′-ACTACCACTGGAAAATACAGAAGTT-3′ (targeting exon intron2-exon3 junction)

*Stab1-Ex4-Mo:*

5′-ACAGCTGACAGAAGAATATCGTTTT-3′ (targeting intron3-exon4 junction)

*HOXC9-TB-Mo:*

5′-GTTTCCCTTCTCTTTTACATGCATC-3′ (1 base 5′ of ATG)

*Standard Control-Mo (Co-Mo)*

For *in vivo rescue* experiments, HOXC9 mRNA was generated. The zebrafish full length clone (ID: 7255006) containing the complete HOXC9 cDNA in the plasmid pME18S-FL3 was purchased from OpenBiosystems. HOXC9 cDNA was subcloned by using XhoI (Fermentas) into the pCS2^+^ vector containing a SP6 promoter region, which was a gift from Dirk Meyer (University of Innsbruck). The pCS2^+^ plasmid containing HOXC9 cDNA was then linearized with NotI and mRNA was generated using the mMESSAGE mMACHINE SP6 kit (Ambion) according to the manufacturer’s protocol. The mRNA was diluted in 0,1M KCl to a concentration of 50 ng/µl and injected as described.

### Whole Mount Antibody Staining

For whole mount antibody stainings *tg(fli1:EGFP)* embryos were fixed 2 h in 4% PFA/PBS, washed, dehydrated in methanol and stored at −20°C. Embryos were rehydrated, permeabilized with proteinase K (Macherey-Nagel) and fixed again with 4% PFA/PBS. After blocking in 1% BSA plus 2% serum in PBST, embryos were incubated with an anti-GFP antibody at 4°C overnight. On the following day embryos were washed for 6 h and the secondary antibody was added at 4°C overnight. The colour reaction was developed using the Vectastain ABC kit with horseradish peroxidase and DAB as a chromogen.

### Statistical Analysis and Quantification

Results are expressed as mean ±SD. Comparisons between groups were analyzed by Student’s t-test (two-sided). P values <0.05 were considered as statistically significant. For quantification of zebrafish, *tg(fli1:EGFP)* embryos were stained using an anti-GFP antibody with a DAB-based protocol and analyzed under the microscope. For parachordal angioblast quantification embryos were divided in three groups according to the presence, partial or complete absence of the parachordal lymphangioblasts. The percentage of each group is shown for the several samples in the graph. The total number of counted embryos is shown in the graphs. Defects of the thoracic duct (TD) were observed under the microscope and considered as complete or partial absence of the TD.

### Microscopy and Analysis

For *in vivo* imaging, EGFP-expressing *tg(fli1:EGFP)* embryos were manually dechorionated, anesthetized with 0.003% tricaine and embedded in 1% low-temperature melting agarose (Roth). Fluorescence was analyzed using a DMIRE2 confocal microscope with Leica TCS SP2 True Confocal Scanner (Leica Microsystems) and using a DM6000 CS confocal microscope with Leica TCS SP5 II scanner (Leica Microsystems). Stacks were recorded as indicated in the overview image. Overview pictures and whole mount stainings were analyzed using DMI 6000 B fluorescence microscope (Leica). Pictures of HUVEC spheroids were made using Olympus IX50 microscope.

## Results

### HOXC9 Regulates Parachordal Lymphangioblast Assembly via Stabilin 2

**Figure 1:**
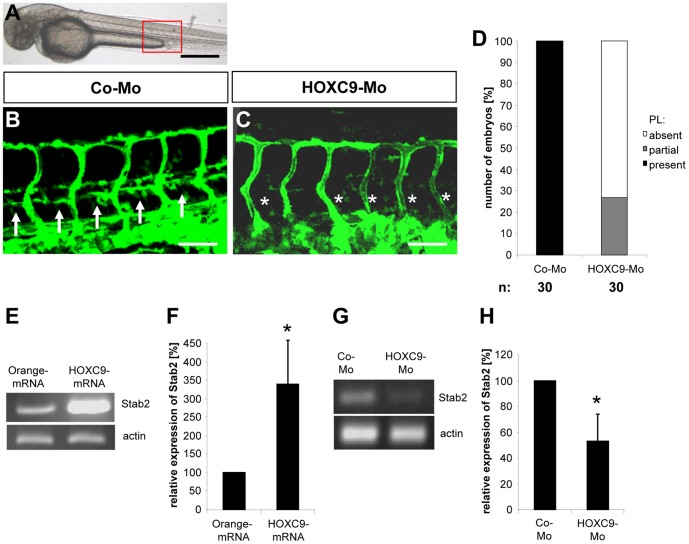
Silencing of HOXC9 expression in zebrafish inhibits assembly of parachordal lymphangioplasts (PLs). (A) Overall morphology of 48 hpf zebrafish embryo after control morpholino injection. Red box shows region displayed in (B) and (C). (B) Normal formation of the PLs (arrows) in 48 hpf *tg(fli1:EGFP*) zebrafish embryo after injection of 4 ng control morpholino. (C) Silencing of HOXC9 expression using 2 ng translational-blocking morpholino disrupted the formation of the PLs (asterisks) in 48 hpf *tg(fli1:EGFP*) zebrafish embryo. (D) Quantification of 48 hpf *tg(fli1:EGFP*) zebrafish embryos showing a disturbed PL formation. Embryos were divided in three groups depending on the PL appearance being completely absent, partially formed or completely present. (E) RT-PCR analysis for increased expression of Stab2 in zebrafish driven by mRNA (50 pg) mediated overexpression of HOXC9. (F) Quantification of (E), n = 3 per group. *p<0.05 vs.Orange-mRNA. (G) RT-PCR analysis for reduced expression of Stab2 in zebrafish driven by morpholino (2 ng) mediated silencing of HOXC9. (H) Quantification of (G), n = 4 per group. *p<0.05 vs.Co-Mo. Black scale bar: 500 µm. White scale bar: 50 µm.

The transcription factor HOXC9 has been reported as an angiogenic regulator which keeps blood vessels in a quiescent state by inhibiting the pro-angiogenic chemokine interleukin 8 (IL-8) [6]. In this study we have addressed the question whether *loss-of-function* experiments for HOXC9 in zebrafish would lead to an altered vascular development. To this end we have performed expression silencing experiments using a recently characterized translational blocking morpholino for HOXC9 [6] in zebrafish embryos. Interestingly, expression silencing of HOXC9 in zebrafish did not induce an aberrant vessel formation as it could be expected from the HOXC9 *gain-of-function* experiments [6] (**Figure 1**). Although vascular development including formation of the dorsal aorta, cardinal vein, intersomitic vessels and the dorsal longitudinal anastomotic vessel in 48 hpf *tg(fli1:EGFP)* HOXC9 morphants was unaltered several HOXC9 morphants showed an impaired assembly of parachordal lymphangioblasts (PLs) at the horizontal myoseptum (**Figure 1** **, A–D and Figure S1**). Since the PLs are finally assembled in zebrafish embryos between 1.8 to 2.2 days postfertilization [28] we also analyzed HOXC9 morphants in 72 hpf zebrafish embryos to exclude a developmental delay. Histological analyses again showed in all HOXC9 morpholino injected zebrafish embryos a partial or complete absence of the PLs while in control morpholino injected zebrafish PLs were completely present (**Figure S2, A–D**). The observed phenotype was quantified by whole mount anti-GFP antibody staining of *tg(fli1:EGFP)* embryos (**Figure S1**) and subsequent analysis under the light microscope. In the 48 hpf stage all embryos in the control-morpholino injected group displayed a complete assembly of PLs whereas 75% of the HOXC9 morphants showed no PLs and 25% only a partial PL assembly (**Figure 1** **, D**). At 72 hpf all HOXC9 morphants displayed only an incomplete or missing PL assembly whereas PL formation was completed in all control morpholino injected embryos (**Figure S2, D**). In conclusion, these experiments identified HOXC9 as an important regulator of PL formation in zebrafish which serves as a precursor of the future thoracic duct.

**Figure 2:**
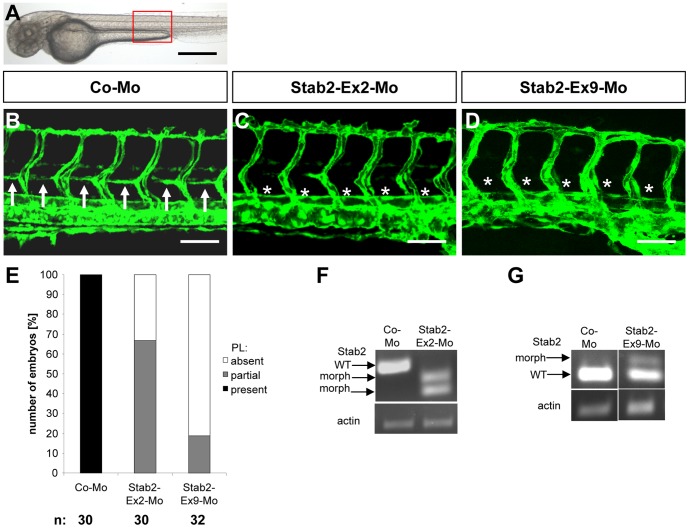
Silencing of Stab2 expression in zebrafish inhibits assembly of parachordal lymphangioplasts (PLs). (A) Overall morphology of 48 hpf zebrafish embryo after control morpholino injection. Red box shows region displayed in (B–D). (B) Normal formation of the PLs (arrows) in 48 hpf *tg(fli1:EGFP*) zebrafish embryo after injection of 4 ng control morpholino. (C,D) Silencing of Stab2 expression using 4 ng splice-blocking morpholino targeting exon 2 (C) or 2 ng splice-blocking morpholino targeting exon 9 (D) disrupted the formation of the PLs (asterisks) in 48 hpf *tg(fli1:EGFP*) zebrafish embryos. (E) Quantification of 48 hpf *tg(fli1:EGFP*) zebrafish embryos showing a disturbed PL formation. Embryos were divided in three groups depending on the PL appearance being completely absent, partially formed or completely present. (F) Functionality of the splice blocking morpholino Stab2-Ex2-Mo. RT-PCR of control morpholino (4 ng) and Stab2-Ex2-Mo (4 ng) injected zebrafish embryos at 24 hpf. (G) Functionality of the splice blocking morpholino Stab2-Ex9-Mo. RT-PCR of control morpholino (4 ng) and Stab2-Ex9-Mo (2 ng) injected zebrafish embryos at 24 hpf. WT: wild type, morph: morphant. Black scale bar: 500 µm. White scale bar: 50 µm.

To identify potential HOXC9 target genes which may promote lymphatic development in zebafish we reanalyzed our recently performed microarray analysis where HOXC9 was overexpressed in HUVECs [6]. In contrast to the strong expression inhibition of IL-8 by HOXC9 [6] we also identified a positive 2.8 fold induction of stabilin 2 expression in HOXC9 overexpressing HUVECs (not shown). In zebrafish, stabilin 2 is strongly expressed in the posterior cardinal vein, which forms the later PLs [18]. Therefore, this expression pattern highlighted stabilin 2 as an attractive candidate of HOXC9 actions, which itself is expressed in the cardinal vein in zebrafish [6]. To analyze stabilin 2′s function on vessel formation in zebrafish we first confirmed HOXC9 as a positive regulator of stabilin 2 expression in zebrafish by overexpressing HOXC9 in zebrafish via mRNA injection. Subsequent analysis of stabilin 2 expression using RT-PCR analysis identified an approximately 3 fold induction of zebrafish stabilin 2 by HOXC9 (**Figure 1** **, E–F**). In addition, complementary HOXC9 *loss-of-function* expression studies in zebrafsh via HOXC9 morpholino injection led to a decreased stabilin 2 expression in zebrafish embryos at 24 hpf (**Figure 1** **, G–H**). Based on the expression and regulation of stabilin 2 by HOXC9 we hypothesized that expression silencing of stabilin 2 in zebrafish will have a similar vascular phenotype as HOXC9 silencing. In order to prove this hypothesis we injected two different splice blocking morpholinos for stabilin 2 in *tg(fli:EGFP)* zebrafish embryos and analyzed formation of the vascular system at 48 hpf and 72 hpf. Indeed formation of PLs was strongly inhibited in both stabilin 2 morphants at 48 hpf and 72 hpf (**Figure 2** **and Figures S1 and S3**). Furthermore, an additional third morpholino targeting the ATG region of the zebrafish stabilin 2 gene displayed a similar vascular phenotype in PL formation (**Figure S4**) indicating that stabilin 2 silencing in zebrafish phenocopies the HOXC9 morphant vascular phenotype.

**Figure 3:**
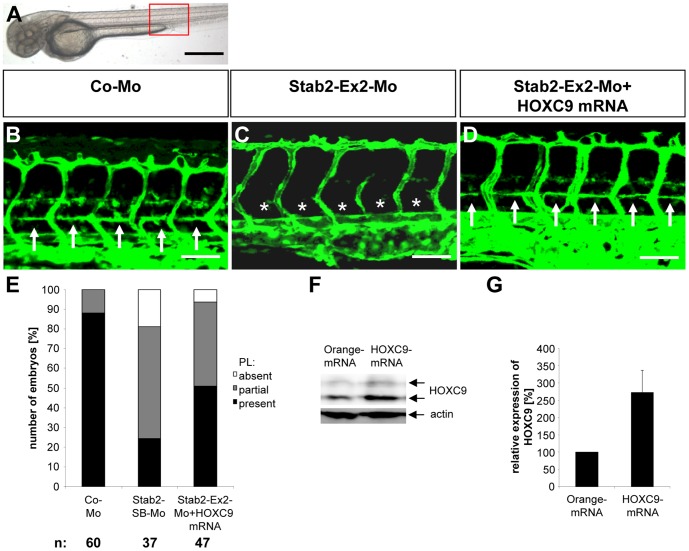
HOXC9 overexpression rescues the defects in parachordal lymphangioplast (PL) formation in Stab2 morphants. (A) Overall morphology of 48 hpf zebrafish embryo after control morpholino injection. Red box shows region displayed in (B–D). (B) Normal formation of the PLs (arrows) in 48 hpf *tg(fli1:EGFP*) zebrafish embryo after injection of 4 ng control morpholino. (C) Silencing of Stab2 expression using 4 ng splice-blocking morpholino disrupted the formation of the PLs (asterisks) in 48 hpf *tg(fli1:EGFP*) zebrafish embryo. (D) Injection of HOXC9 mRNA (50 pg) rescued the Stab2 loss-of-function phenotype in 48 hpf *tg(fli1:EGFP*) zebrafish embryo. (E) Quantification of 48 hpf *tg(fli1:EGFP*) fish embryos showing a disturbed PL formation including rescue experiments using HOXC9 mRNA (50 pg). Embryos were divided in three groups depending on the PL appearance being completely absent, partially formed or completely present. (F) Functionality of the mRNA injection. Western Blot of Orange-mRNA (50 pg) and HOXC9-mRNA (50 pg) injected zebrafish embryos at 24 hpf. (G) Quantification of (F), n = 3 per group. *p<0.05 vs. Orange-mRNA. Black scale bar: 500 µm. White scale bar: 50 µm.

In order to finally prove that HOXC9 mediates its function via stabilin 2 expression, we performed HOXC9 mRNA rescue experiments in zebrafish (**Figure 3** **and Figures S5 and S6**). Injection of HOXC9 mRNA led to an enhanced HOXC9 protein expression in zebrafish embryo as demonstrated by Western blot (**Figure 3** **, F–G**), but HOXC9 mRNA injection alone did not alter formation of the vasculature in zebrafish embryos when low doses of mRNA (50 pg) were used (**Figure S7**). However, high amounts of HOXC9 mRNA (100 pg and higher) led to morphological malformation and vascular defects in zebrafish embryos (data not shown). We then injected the HOXC9 mRNA (50 pg) together with the stabilin 2 splice blocking morpholino Stab2-Ex2-Mo in *tg(fli:EGFP)* zebrafish embryos and found in 48 hpf and 72 hpf zebrafish embryos an almost complete assembly of PLs (**Figure 3** **, A-E and Figure S6, A–E**). Quantification revealed a partial rescue of PL formation after HOXC9 mRNA injection in 48 hpf stabilin 2 morphant embryos (**Figure 3** **, E**). A complete rescue was identified in 72 hpf stabilin 2 morphants where the double injected embryos displayed the same distribution of complete and partial presence of the PLs as the control morpholino injected embryos did (**Figure S6, E**). Together, the data indicated that stabilin 2 is an important regulator of parachordal lymphangioblast assembly during zebrafish development and that stabilin 2 expression is controlled by the transcription factor HOXC9.

### Stabilin 1 Regulates Parachordal Lymphangioblast Assembly Independently of HOXC9

**Figure 4:**
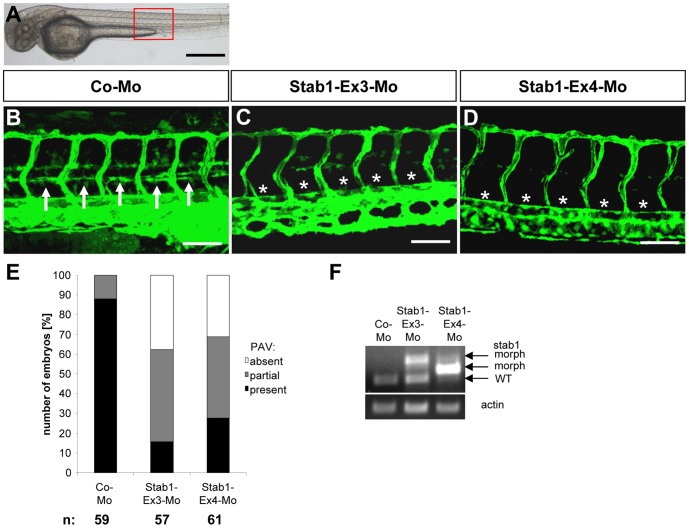
Silencing of Stab1 expression in zebrafish inhibits assembly of parachordal lymphangioplasts (PLs). (A) Overall morphology of 48 hpf zebrafish embryo after control morpholino injection. Red box shows region displayed in (B–D). (B) Normal formation of the PLs (arrows) in 48 hpf *tg(fli1:EGFP*) zebrafish embryo after injection of 4 ng control morpholino. (C,D) Silencing of Stab1 expression using 4 ng splice-blocking morpholino targeting exon 3 (C) or 12 ng splice-blocking morpholino targeting exon 4 (D) disrupted the formation of the PLs (asterisks) in 48 hpf *tg(fli1:EGFP*) fish embryos. (E) Quantification of 48 hpf *tg(fli1:EGFP*) fish embryos showing a disturbed PL formation. Embryos were divided in three groups depending on the PL appearance being completely absent, partially formed or completely present. (F) Functionality of the splice blocking morpholinos Stab1-Ex3-Mo and Stab1-Ex4-Mo. RT-PCR of control morpholino (4 ng), Stab1-Ex3-Mo (4 ng) and Stab1-Ex4-Mo (12 ng) injected zebrafish embryos at 24 hpf. WT: wild type, morph: morphant. Black scale bar: 500 µm. White scale bar: 50 µm.

**Figure 5:**
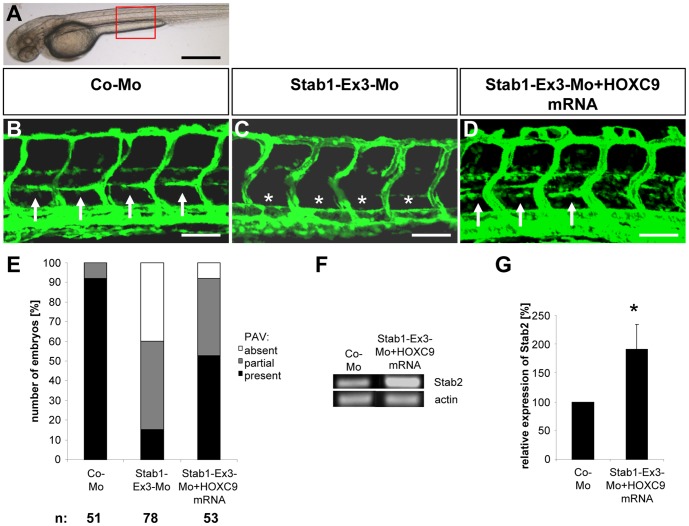
HOXC9 overexpression rescues the defects in parachordal lymphangioplast (PL) formation in Stab1 morphants. (A) Overall morphology of 48 hpf zebrafish embryo after control morpholino injection. Red box shows region displayed in (B–D). (B) Normal formation of the PLs (arrows) in 48 hpf *tg(fli1:EGFP*) fish embryo after injection of 4 ng control morpholino. (C) Silencing of Stab1 expression using 4 ng splice-blocking morpholino disrupted the formation of the PLs (asterisks) in 48 hpf *tg(fli1:EGFP*) zebrafish embryo. (D) Injection of HOXC9 mRNA (50 pg) rescued the Stab1 loss-of-function phenotype in 48 hpf *tg(fli1:EGFP*) zebrafish embryo. (E) Quantification of 48 hpf *tg(fli1:EGFP*) zebrafish embryos showing a disturbed PL formation including rescue experiments using HOXC9 mRNA (50 pg). Embryos were divided in three groups depending on the PL appearance being completely absent, partially formed or completely present. (F) RT-PCR analysis for increased expression of Stab2 in zebrafish injected with the Stab1-Ex3-Mo (4 ng) and HOXC9 mRNA (50 pg). (G) Quantification of (F), n = 3 per group. *p<0.05 vs. Co-Mo. Black scale bar: 500 µm. White scale bar: 50 µm.

Stabilin 1 and stabilin 2 are both members of the family of fasciclin-like hyaluronan receptor homologues and have similar functions [9]. Thus we investigated the role of stabilin 1 on PL development in vascular development in zebrafish (**Figure 4** **and Figures S1 and S8**). Using two different splice blocking morpholinos directed against stabilin 1 (**Figure 4** **, F**) a similar vascular phenotype like in stabilin 2 morphants was observed showing that the assembly of PLs was strongly inhibited in *tg(fli:EGFP)* zebrafish embryos (**Figure 4** **, A-E and Figure S1, E-F′**). PL assembly was partial or completely absent in both stabilin 1 morphants at 48 hpf whereas control morpholino injected embryos mostly displayed a complete PL assembly (**Figure 4** **, B,E**). At 72 hpf PL formation in more than half of the stabilin 1 morphant embryos was impaired (**Figure S8, E**). Next, we have addressed the question if HOXC9 regulates stabilin 1 mediated PL regulation in zebrafish although the endothelial HOXC9 expression data in HUVECs did not show a HOXC9 mediated increased expression of stabilin 1 (not shown). Surprisingly, injection of HOXC9 mRNA together with the stabilin 1 morpholino Stab1-Ex3-Mo displayed a rescue effect on PL assembly at 48 hpf and 72 hpf, which led to a normal PL assembly compared to the disrupted PLs of stabilin 1 morphants (**Figure 5** **, A-E, Figure S5, D–E′ and Figure S9, A–E**). HOXC9 expression partially compensated the PL defect of stabilin 1 *tg(fli:EGFP)* morphants at 48 hpf (**Figure 5** **, E**) and almost completely at 72 hpf (**Figure S9, E**). This was not entirely expected because stabilin 1 was not regulated by HOXC9 in HUVECs [6] and furthermore, we could not observe an expression regulation of stabilin 1 by HOXC9 in zebrafish (**Figure S7, G,H**). The rescue effect however, could be explained by an enhanced expression of stabilin2 in the HOXC9 mRNA injected stabilin1 morphants (**Figure 5** **, F–G**) which suggested that stabilin 2 may have a compensatory effect in the stabilin 1 *loss-of-function* experiments. Yet, we could not observe a synergistic vascular effect of a double knockdown of both stabilins in zebrafish embryos (**Figure S10**). The vascular effects on PL formation of both stabilin 1 and stabilin 2 are comparable to the single *loss-of-function* experiments of either stabilin 1 or stabilin 2. Therefore the data indicate that stabilin 1 acts as a PL assembly regulator during zebrafish development, which is independent of HOXC9 and that its function is congruent with stabilin 2.

### Silencing of HOXC9, Stabilin 2 and Stabilin 1 Leads to Thoracic Duct Formation Defects

**Figure 6:**
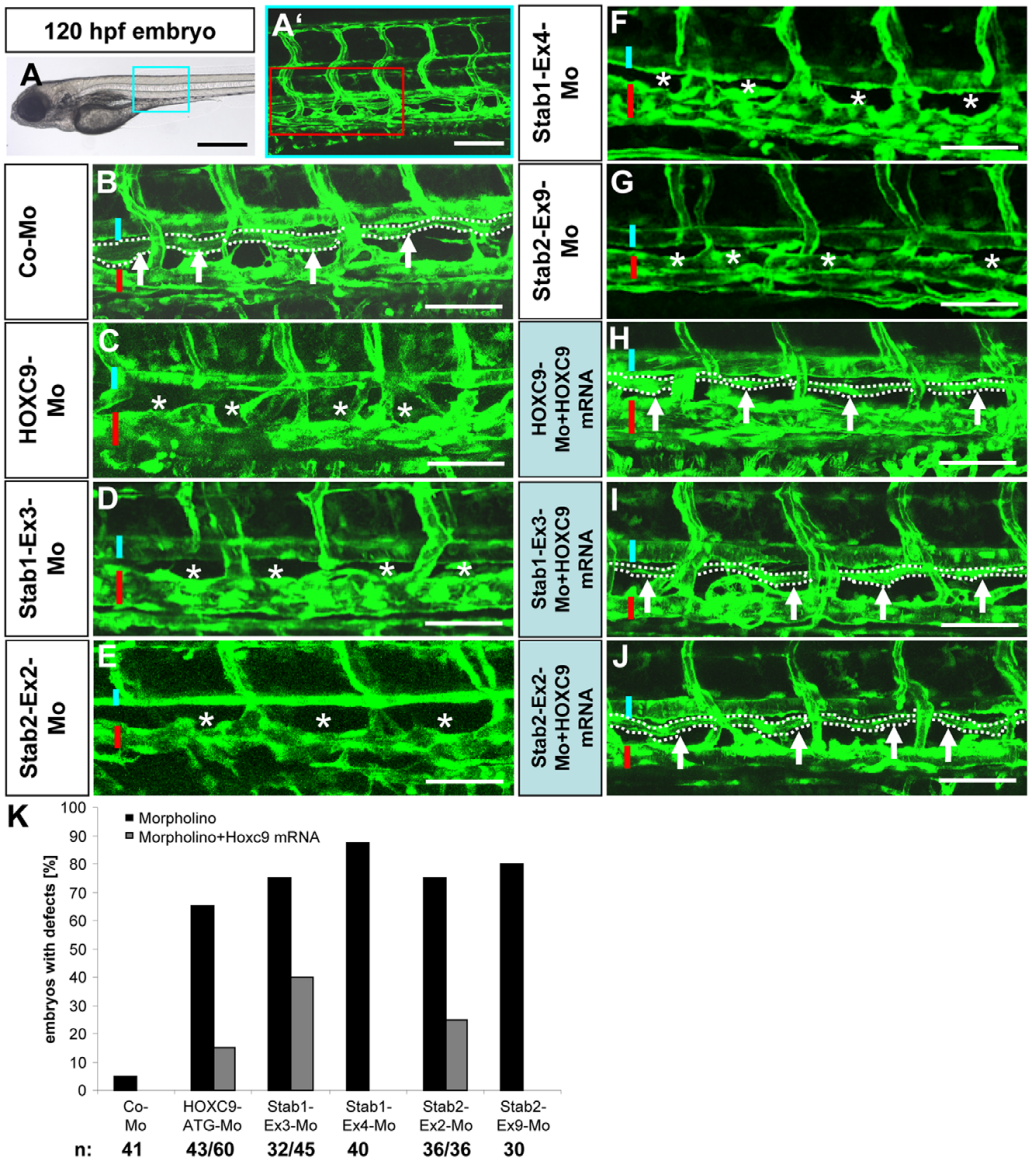
Silencing of HOXC9, Stab1 and Stab2 expression in zebrafish inhibits formation of the thoracic duct (TD). (A) 120 hpf *tg(fli1:EGFP*) zebrafish embryo. Blue box marks the region magnified in (A′). (A′) Trunk vasculature of the embryo shown in (A). Red box marks the region magnified in (B–J). (B) Normal formation of the TD (arrows and dotted line) in 120 hpf *tg(fli1:EGFP*) zebrafish embryos after injection of control morpholino. Blue bar marks dorsal aorta and red bar marks cardinal vein. (C–G) Silencing of HOXC9, Stab1 and Stab2 expression using the indicated morpholinos disrupted the formation of the TD (asterisks) in 120 hpf *tg(fli1:EGFP*) zebrafish embryos. (H–J) Co-injection of 50 pg HOXC9 mRNA rescued the defects in TD formation caused by silencing of HOXC9, Stab1 and Stab2 using the indicated morpholinos. (K) Quantification of defects in TD formation of embryos shown in (A–J) including rescue experiments with 50 pg HOXC9-mRNA. Black scale bar: 500 µm. White scale bar: 50 µm.

During zebrafish lymphangiogenesis sprouts from the posterior cardinal vein give rise to the parachordal lymphangioblasts which assemble in the horizontal myoseptum. The PLs later migrate and proliferate ventral from the dorsal aorta and become lymphatic endothelial cells which form the thoracic duct (TD) at 120 hpf [19], [20]. Thus we hypothesized that TD formation is affected by silencing HOXC9, stabilin 2 or stabilin 1 in zebrafish. Indeed, in all HOXC9, stabilin 2 and stabilin 1 zebrafish morphants we observed a strong inhibition on TD formation compared to the control morpholino injected zebrafish (**Figure 6** **, A-G,K**). The number of zebrafish embryos which displayed a complete loss or a scarcely formed TD ranged between 65% and 90% in the HOXC9, stabilin 2 and stabilin 1 morphants compared to the control zebrafish embryos at 120 hpf (5%) (**Figure 6** **, K**) Furthermore a rescue effect on PL formation after HOXC9 (HOXC9-ATG-Mo), stabilin 2 (Stab2-Ex2-Mo) or stabilin 1 (Stab1-Ex3-Mo) morpholino injection together with HOXC9 mRNA injection was observed (**Figure 6** **, H–K**). In conclusion the zebrafish data suggest that HOXC9, stabilin 2 and stabilin 1 are important regulators of PL and TD formation in zebrafish.

### Stabilin 2 and Stabilin 1 Control Endothelial Cell Migration and In-gel Sprouting Angiogenesis *in vitro*

**Figure 7:**
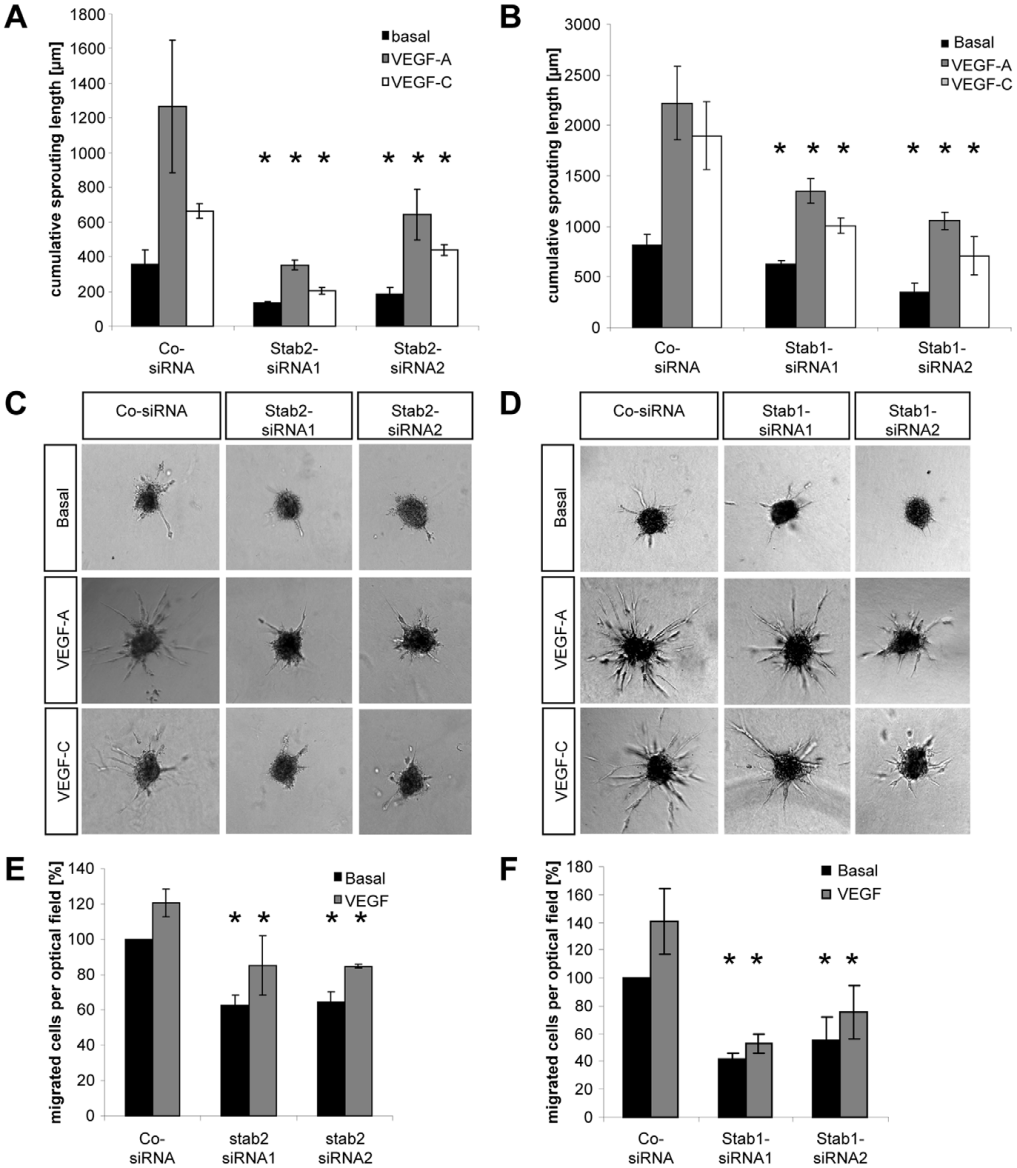
Silencing of Stab2 or Stab1 expression in endothelial cells inhibits endothelial sprouting and migration. (A,B) siRNA mediated silencing of Stab2 (A) or Stab1 (B) inhibited basal, VEGF-A and VEGF-C driven in-gel sprouting in HUVECs. (C,D) Representative spheroids from the sprouting assay shown in (A,B). (E,F) Silencing of Stab2 (E) or Stab1 (F) in HUVECs inhibited basal and VEGF-A driven endothelial migration in the modified Boyden chamber assay. HUVECs were transfected with two different Stab2 or Stab1 siRNAs and allowed to migrate through a membrane for 3 h using 25 ng/ml VEGF-A. Migrated cells were stained and counted under the microscope. n = 3 per group. *p<0.05 vs. control siRNA.

Sprouting and migration are important steps during lymphatic development [2]. Thus we have used HUVECs as venous endothelial cells and addressed the question if stabilin 2 and stabilin 1 regulate these cellular processes *in vitro* (**Figure 7** **and Figure S11**). To this end HUVEC spheroids lacking stabilin 2 or stabilin 1 expression (**Figure S11, A–B′**) were embedded in collagen and subsequent in-gel sprouting angiogenesis was analyzed. To stimulate in-gel sprouting VEGF-A as well as VEGF-C were used as important stimulators of lymphangiogenesis [1]. Two different siRNAs directed against stabilin 2 as well as against stabilin 1 were able to reduce basal endothelial sprouting. In addition, VEGF-A, VEGF-C (**Figure 7** **, A–D**) or FCS (data not shown) induced sprouting in stabilin 2 and stabilin 1 deficient HUVEC spheroids were also significantly reduced. Similarly we tested endothelial cell migration in a modified Boyden chamber assay and found that silencing of stabilin 2 or stabilin 1 reduced basal and VEGF-A driven endothelial cell migration (**Figure 7** **, E–F**). Likewise, analysis of cytoskeletal reorganization by phalloidin staining in stabilin 2 and stabilin 1 silenced HUVECs upon VEGF stimulation showed reduced and slowed actin synthesis which further supports reduced cell motility in stabilin 2 and stabilin 1 deficient endothelial cells (**Figure 12**). Lastly, we analyzed VEGF-A driven Akt phosphorylation in stabilin 2 silenced HUVECs and observed a reduced basal and VEGF-A stimulated Akt phosphorylation which further suggests that stabilin 2 function is not exclusively restricted to VEGF-A signaling (**Figure 13**).To exclude the possibility that the negative effects of stabilin 2 and stabilin 1 silencing on sprouting and migration are driven by enhanced apoptosis, we performed apoptosis assays in stabilin 2 and stabilin 1 deficient HUVECs. Yet, none of the used siRNAs enhanced caspase3/7 activity in HUVECs (**Figure S11, E–F**). Finally, we have analyzed HOXC9 transduced and HOXC9 silenced HUVECs for stabilin 2 and stabilin 1 expression. As expected and similar to zebrafish, stabilin 2 expression is upregulated in HOXC9 transduced HUVECs (**Figure S11, C–C′**) whereas its expression was decreased in HOXC9 silenced HUVECs (**Figure S11, D,D′**). In contrast and similar to zebrafish, stabilin 1 expression is not regulated in HOXC9 *loss-of-function* and *gain-of-function* experiments in HUVECs (not shown).

All together, the *in vitro* data complement the findings we have observed in zebrafish regarding the regulative role of stabilin 2 and stabilin 1 during zebrafish lymphatic development. They demonstrate that stabilin 2 and stabilin 1 control venous endothelial sprouting and migration and thus identified an important mechanism for the developing lymphatic system.

## Discussion

The transcription factor HOXC9 was identified in this study as a novel regulator of lymphangiogenesis in zebrafish that acts via regulation of the hyaluronan receptor stabilin 2. This was demonstrated by the finding that (i) HOXC9 silencing in zebrafish leads to impaired parachordal lymphangioblast assembly and thoracic duct formation, (ii) HOXC9 regulates stabilin 2 expression in zebrafish and in cultured endothelial cells, (iii) stabilin 2 and stabilin 1 expression silencing phenocopies the HOXC9 morphant vascular phenotype in zebrafish and (iv) forced expression of HOXC9 compensates the stabilin 2 and stabilin 1 phenotype in zebrafish.

It is now ten years ago since the homeobox transcription factor Prox1 was identified as a master regulator in lymphangiogenesis, that is sufficient to induce a lymphatic differentiation in blood vascular endothelial cells [29], [30]. The complete loss of the lymphatic system in Prox1 knockout mice emphasizes its importance [31]. As a master switch, Prox1 induces lymphatic genes, such as VEGFR3 and the lymphatic marker podoplanin, whereas it downregulates blood vascular specific genes [29], [30]. In our previous study we discovered HOXC9 as an inhibitor of the pro-angiogenic chemokine IL-8, which leads to quiescence in blood endothelial cells [6]. The transcriptome analyses performed in this previous study furthermore revealed a positive regulation for the lymphatic marker lyve1 (2.6 fold increase) and for stabilin 2 (2.8 fold increase). Similar to Prox1, HOXC9 can suppress pro-angiogenic factor, whereas it stimulates pro-lymphangiogenic genes. As the HOXC9 expression data showed no regulation of other major lymphatic transcriptional regulators such as Prox1 or Sox18, HOXC9 probably acts downstream of these molecules. According to our data HOXC9 upregulates the hyaluronan receptor stabilin 2 which enables migration and sprouting of lymphatic progenitors within the venous endothelial cells. Since HOXC9 and stabilin 2 are both expressed in the cardinal vein in zebrafish [6], [18] and experiments in HUVECs showed HOXC9 driven expression of stabilin 2 and a direct effect of stabilin 2 on endothelial cell function, the data imply a cell-autonomous function for HOXC9 and stabilin 2 in the vasculature. We also analyzed the upstream region of the stabilin 2 gene for putative HOX binding sites (**Figure S14**) and identified several possible binding sites, which were previously described [32]. This indicates that HOXC9 may bind to the stabilin 2 promoter region and regulates stabilin 2 expression directly. Yet, further studies need to be performed to support this hypothesis.

It has been reported that stabilin 2 can also interact with integrin [15] and is involved in cell aggregation [33], thus stabilin 2 could also enhance cell-cell contacts and cell adhesion during lymphatic development. Therefore, our findings demonstrate that in addition to stabilin 2′s clearance function for heparin or hyaluronan [17], it also acts as a physiological regulator of lymphangiogenesis in zebrafish. Interestingly, lyve1, which is also upregulated by HOXC9, has a similar function like stabilin 2 acting as a hyaluronan receptor. Yet, the physiological function of lyve1 is still unclear since the lyve1 knockout mouse revealed no obvious developmental lymphatic phenotype which could be due to compensatory expression of other hyaluronan receptors [34].

Stabilin 1, which is a closely related hyaluronan receptor homolog to stabilin 2, acts in a similar way like stabilin 2. We could show that silencing of stabilin 1 in zebrafish leads to impaired parachordal lymphangioblast assembly and thoracic duct formation but stabilin 1′s function is independent of HOXC9. Yet, similar to stabilin 2, stabilin 1 regulates migration and sprouting of human umbilical vein endothelial cells. It has already been suggested in earlier studies that stabilin 1 has angiogenesis modulating effects by mediating cell-cell interaction in HUVECs [11]. In contrast to stabilin 2, stabilin 1 is also expressed in lymphatic vessels and in macrophages [13]. This supports an additional important function for stabilin 1 because macrophages play an important role in vessel anastomosis via tip cell fusion [35]. This may suggest that stabilin 1 regulates not only endothelial cell migration and sprouting in early lymphatic development but acts also in later stages of lymphatic vessel formation during vessel anastomosis. Surprisingly, the vascular phenotype in the stabilin 1 zebrafish morphants could be rescued by HOXC9 overexpression although stabilin 1 was not regulated by HOXC9. This is explained by the HOXC9 induced upregulation of stabilin 2, which can compensate the loss of stabilin 1. However, we have observed no synergistic effects on lymphatic development in the stabilin 1/stabilin 2 double morphants which argues that stabilin 1 and stabilin 2 act in a similar cellular process during lymphatic development.

Based on this study and our previous work we suggest the following model for HOXC9’s function in the vasculature. HOXC9 is expressed in the cardinal vein in zebrafish and keeps venous endothelial cells via inhibition of IL-8 in a quiescent state. In early stages of lymphatic differentiation HOXC9 induces expression of the hyaluronan receptor stabilin 2. Together with stabilin 1, both receptors mediate lymphangiogenesis via endothelial cell migration, sprouting, cell-cell adhesion and recruitment of macrophages. Notably, at this stage the inhibition of IL-8 by HOXC9 can enhance the activity of Prox1, which was reported to be inhibited by IL-8 [36]. Thus, HOXC9 contributes to lymphatic differentiation via enhancement of Prox1 function in addition to the induction of stabilin 2 expression.

In conclusion this study characterized HOXC9, stabilin 2 and stabilin 1 as three novel regulators of lymphatic vascular development and their functional interaction in zebrafish and in endothelial cells. The findings for HOXC9, stabilin 2 and stabilin 1 strongly contribute to our understanding of the molecular pathways and mechanisms during lymphangiogenesis and can also be beneficial in the understanding of disease processes such as inflammation, edema formation, tumor development and metastasis, where lymphangiogenesis is involved.

## Supporting Information

Figure S1 **Silencing of HOXC9, Stab2 and Stab1 expression in zebrafish inhibits assembly of parachordal lymphangioplasts (PLs).** (A–F′) Whole mount antibody staining against GFP in 48 hpf (A–F) and 72 h hpf (A′–F′) *tg(fli1:EGFP*) zebrafish embryos injected with the indicated morpholinos. (A,A′) Normal formation of the PLs (arrows) in 48 hpf (A) and 72 hpf (A′) *tg(fli1:EGFP*) zebrafish embryos after injection of 4 ng control morpholino. (B–F′) Silencing of HOXC9, Stab2 and Stab1 expression using the indicated morpholinos disrupted formation of the PLs (asterisks) in 48 hpf (B–F) and 72 hpf (B′–F′) *tg(fli1:EGFP*) zebrafish embryos. Black scale bar: 100 µm.
(JPG)

Figure S2 **Silencing of HOXC9 expression in zebrafish inhibits assembly of parachordal lymphangioplasts (PLs) at 72 hpf.** (A) Overall morphology of 72 hpf zebrafish embryo after control morpholino injection. Red box shows region displayed in (B) and (C). (B) Normal formation of the PLs (arrows) in 72 hpf *tg(fli1:EGFP*) zebrafish embryo after injection of 4 ng control morpholino. (C) Silencing of HOXC9 expression using 2 ng translational-blocking morpholino disrupted the formation of the PLs (asterisks) in 72 hpf *tg(fli1:EGFP*) zebrafish embryo. (D) Quantification of 72 hpf *tg(fli1:EGFP*) zebrafish embryos showing a disturbed PL formation. Embryos were divided in three groups depending on the PL appearance being completely absent, partially formed or completely present. Black scale bar: 500 µm. White scale bar: 50 µm.
(JPG)

Figure S3 **Silencing of Stab2 expression in zebrafish inhibits assembly of parachordal lymphangioplasts (PLs) at 72 hpf.** (A) Overall morphology of 72 hpf zebrafish embryo after control morpholino injection. Red box shows region displayed in (B–D). (B) Normal formation of the PLs (arrows) in 72 hpf *tg(fli1:EGFP*) zebrafish embryo after injection of 4 ng control morpholino. (C,D) Silencing of Stab2 expression using 4 ng splice-blocking morpholino targeting exon 2 (C) or 2 ng splice-blocking morpholino targeting exon 9 (D) disrupted the formation of the PLs (asterisks) in 72 hpf *tg(fli1:EGFP*) zebrafish embryos. (E) Quantification of 72 hpf *tg(fli1:EGFP*) zebrafish embryos showing a disturbed PL formation. Embryos were divided in three groups depending on the PL appearance being completely absent, partially formed or completely present. Black scale bar: 500 µm. White scale bar: 50 µm.
(JPG)

Figure S4 **Silencing of Stab2 expression using an ATG-morpholino in zebrafish inhibits assembly of parachordal lymphangioplasts (PLs).** (A;D) Overall morphology of 48 hpf (A) and 72 hpf (D) zebrafish embryos after injection of control morpholino. Red box shows region displayed below in (B,C,E,F). (B,E) Normal formation of the PLs (arrows) in 48 hpf (B) and 72 hpf (E) *tg(fli1:EGFP*) zebrafish embryos after injection of 4 ng control morpholino. (C,F) Silencing of Stab2 expression using 2 ng translational-blocking morpholino disrupted the formation of the PLs (asterisks) in 48 hpf (C) and 72 hpf (F) *tg(fli1:EGFP*) zebrafish embryos. (G,H) Quantification of 48 hpf (G) and 72 hpf (H) *tg(fli1:EGFP*) zebrafish embryos showing a disturbed PL formation. Embryos were divided in three groups depending on the PL appearance being completely absent, partially formed or completely present. Black scale bar: 500 µm. White scale bar: 50 µm.
(JPG)

Figure S5 **HOXC9 overexpression rescues defects in parachordal lymphangioplast (PL) formation in Stab2 and Stab1 morphants.** (A–E′) Whole mount antibody staining against GFP in 48 hpf (A–E) and 72 hpf (A′–E′) *tg(fli1:EGFP*) zebrafish embryos injected with the indicated morpholinos. (A,A′) Normal formation of the PLs (arrows) in 48 hpf (A) and 72 hpf (A′) *tg(fli1:EGFP*) zebrafish embryos after injection of 4 ng control morpholino. (B,B′) Silencing of Stab2 expression using 4 ng Stab2-Ex2-Mo disrupted formation of the PLs (asterisks) in 48 hpf (B) and 72 hpf (B′) *tg(fli1:EGFP*) zebrafish embryos. (C,C′) Injection of HOXC9 mRNA (50 pg) rescued the Stab2 loss-of-function phenotype in 48 hpf (C) and 72 hpf (C′) *tg(fli1:EGFP*) zebrafish embryos. (D,D′) Silencing of Stab1 expression using 4 ng Stab1-Ex3-Mo disrupted formation of the PLs (asterisks) in 48 hpf (D) and 72 hpf (D′) *tg(fli1:EGFP*) zebrafish embryos. (E,E′) Injection of HOXC9 mRNA (50 pg) rescued the Stab1 loss-of-function phenotype in 48 hpf (E) and 72 hpf (E′) *tg(fli1:EGFP*) zebrafish embryos. Black scale bar: 500 µm.
(JPG)

Figure S6 **HOXC9 overexpression rescues the defects in parachordal lymphangioplast (PL) formation in Stab2 morphants at 72 hpf.** (A) Overall morphology of 72 hpf zebrafish embryo after control morpholino injection. Red box shows region displayed in (B–D). (B) Normal formation of the PLs (arrows) in 72 hpf *tg(fli1:EGFP*) zebrafish embryo after injection of 4 ng control morpholino. (C) Silencing of Stab2 expression using 4 ng splice-blocking morpholino disrupted the formation of the PLs (asterisks) in 72 hpf *tg(fli1:EGFP*) zebrafish embryo. (D) Injection of HOXC9 mRNA (50 pg) rescued the Stab2 loss-of-function phenotype in 72 hpf *tg(fli1:EGFP*) zebrafish embryo. (E) Quantification of 72 hpf *tg(fli1:EGFP*) fish embryos showing a disturbed PL formation including rescue experiments using HOXC9 mRNA (50 pg). Embryos were divided in three groups depending on the PL appearance being completely absent, partially formed or completely present. Black scale bar: 500 µm. White scale bar: 50 µm.
(JPG)

Figure S7 **Low dose injection (50** **pg) of HOXC9 mRNA shows no effect on zebrafish vascular morphology.** (A,D) Overall morphology of 48 hpf (A) and 72 hpf (D) zebrafish embryos after injection of control morpholino. Red box shows region displayed in (B,C,E,F). (B,E) Normal formation of the PLs (arrows) in 48 hpf (B) and 72 hpf (E) *tg(fli1:EGFP*) fish embryos after injection of 4 ng control morpholino or 4 ng control morpholino combined with 50 pg HOXC9 mRNA (C,F). (G) RT-PCR analysis of zebrafish lysates injected with indicated morpholinos showed no regulation of stabilin 1 by HOXC9 silencing. (H) RT-PCR analysis of zebrafish lysates injected with indicated mRNA showed no regulation of stabilin 1 by HOXC9 overexpression. Black scale bar: 500 µm. White scale bar: 50 µm.
(JPG)

Figure S8 **Silencing of Stab1 expression in zebrafish inhibits assembly of parachordal lymphangioplasts (PLs) at 72 hpf.** (A) Overall morphology of 72 hpf zebrafish embryo after control morpholino injection. Red box shows region displayed in (B–D). (B) Normal formation of the PLs (arrows) in 72 hpf *tg(fli1:EGFP*) zebrafish embryo after injection of 4 ng control morpholino. (C,D) Silencing of Stab1 expression using 4 ng splice-blocking morpholino targeting exon 3 (C) or 12 ng splice-blocking morpholino targeting exon 4 (D) disrupted the formation of the PLs (asterisks) in 72 hpf *tg(fli1:EGFP*) fish embryos. (E) Quantification of 72 hpf *tg(fli1:EGFP*) fish embryos showing a disturbed PL formation. Embryos were divided in three groups depending on the PL appearance being completely absent, partially formed or completely present. Black scale bar: 500 µm. White scale bar: 50 µm.
(JPG)

Figure S9 **HOXC9 overexpression rescues the defects in parachordal lymphangioplast (PL) formation in Stab1 morphants.** (A) Overall morphology of 72 hpf zebrafish embryo after control morpholino injection. Red box shows region displayed in (B–D). (B) Normal formation of the PLs (arrows) in 72 hpf *tg(fli1:EGFP*) fish embryo after injection of 4 ng control morpholino. (C) Silencing of Stab1 expression using 4 ng splice-blocking morpholino disrupted the formation of the PLs (asterisks) in 72 hpf *tg(fli1:EGFP*) zebrafish embryo. (D) Injection of HOXC9 mRNA (50 pg) rescued the Stab1 loss-of-function phenotype in 72 hpf *tg(fli1:EGFP*) zebrafish embryo. (E) Quantification of 72 hpf *tg(fli1:EGFP*) zebrafish embryos showing a disturbed PL formation including rescue experiments using HOXC9 mRNA (50 pg). Embryos were divided in three groups depending on the PL appearance being completely absent, partially formed or completely present. Black scale bar: 500 µm. White scale bar: 50 µm.
(JPG)

Figure S10 **Expression silencing of Stab1 and Stab2 shows no additive effect on parachordal lymphangioplast (PL) assembly.** (A,D) Overall morphology of 48 hpf (A) and 72 hpf (D) zebrafish embryos after injection of control morpholino. Red box shows region displayed in (B,C,E,F). (B,E) Normal formation of the PLs (arrows) in 48 hpf (B) and 72 hpf (E) *tg(fli1:EGFP*) zebrafish embryos after injection of 4 ng control morpholino. (C,F) Double silencing of Stab1 and Stab2 expression using 4 ng of each splice-blocking morpholino for Stab1 and Stab2, respectively, disrupted formation of the PLs (asterisks) in 48 hpf (C) and 72 hpf (F) *tg(fli1:EGFP*) zebrafish embryos. (G,H) Quantification of 48 hpf (G) and 72 hpf (H) *tg(fli1:EGFP*) zebrafish embryos showing a disturbed PL formation. Embryos were divided in three groups depending on the PL appearance being completely absent, partially formed or completely present. Black scale bar: 500 µm. White scale bar: 50 µm.
(JPG)

Figure S11 **Silencing and regulation of Stab2 and Stab1 expression in endothelial cells.** (A,B) Functionality of the Stab2 (A) and Stab1 (B) siRNAs. RT-PCR (A) and Western Blot (B) of HUVECs transfected with control siRNA or two different Stab2 or Stab1 siRNA. (A′,B′) Quantification of (A,B) n = 3 per group. *p<0.05 vs. control siRNA. (C) RT-PCR analysis for increased expression of Stab2 in HUVECs driven by adenovirus mediated overexpression of HOXC9. (C′) Quantification of (C), n = 3 per group. *p<0.05 vs. Ad-Cherry. (D) RT-PCR analysis for reduced expression of Stab2 in HUVECs driven by siRNA mediated silencing of HOXC9. (D′) Quantification of (D), n = 3 per group. *p<0.05 vs. Co-siRNA. (E,F) Stab2 (E) or Stab1 (F) siRNAs did not induce apoptosis in HUVECs as measured by caspase 3/7 activity. As a positive control, apoptosis in control siRNA transfected HUVECs was induced by staurosporin (n = 3 per group, *p<0.05 vs. control siRNA).
(JPG)

Figure S12 **Basal and VEGF-A induced actin filament synthesis in HUVECs.** HUVECs were transfected with control-, stab2- and stab1-siRNA. 48 hours after transfection, HUVECs were stimulated with VEGF-A for the indicated time points and cells were subsequently stained with phalloidin-Alexa-546. All images were captured for 100 ms. Scale bar: 100 µm.
(JPG)

Figure S13 **Silencing of Stab2 leads to decreased basal and VEGF-driven Akt phosphorylation in HUVECs.** (A) Western blot analysis showing phosphorylated Akt (pAkt) and total Akt (tAkt). HUVECs were transfected with control- or Stab2-siRNA and subsequently stimulated with VEGF. (B) Quantification of (A), n = 3 per group.
(JPG)

Figure S14 **Upstream region of stabilin 2 gene with putative HOX binding sites.** The region 2 kb upstream of exon 1 is displayed. The untranslated region upstream of ATG (underlined) is marked in violet. Putative HOX binding sites are highlighted in red.
(JPG)
