# Supplementary figures and images for: HOXC9 Regulates Formation of Parachordal Lymphangioplasts and the Thoracic Duct in Zebrafish via Stabilin 2
Source: PLoS One. 2013 Mar 6;8(3):e58311. doi: 10.1371/journal.pone.0058311 (PMC3590145; doi:10.1371/journal.pone.0058311)

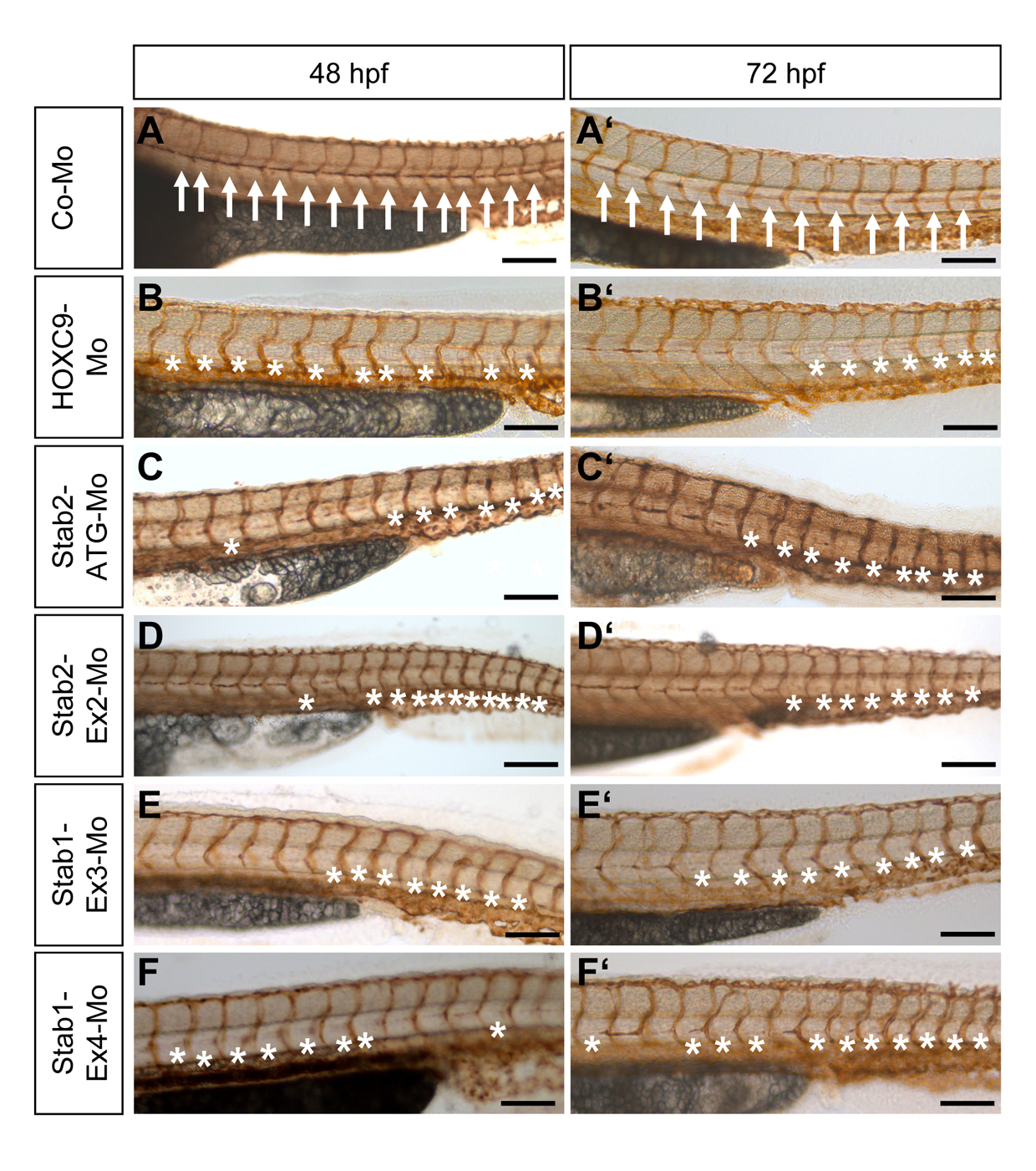

Supplement: Figure S1 — Silencing of HOXC9, Stab2 and Stab1 expression in zebrafish inhibits assembly of parachordal lymphangioplasts (PLs). (A–F′) Whole mount antibody staining against GFP in 48 hpf (A–F) and 72 h hpf (A′–F′) tg(fli1:EGFP) zebrafish embryos injected with the indicated morpholinos. (A,A′) Normal formation of the PLs (arrows) in 48 hpf (A) and 72 hpf (A′) tg(fli1:EGFP) zebrafish embryos after injection of 4 ng control morpholino. (B–F′) Silencing of HOXC9, Stab2 and Stab1 expression using the indicated morpholinos disrupted formation of the PLs (asterisks) in 48 hpf (B–F) and 72 hpf (B′–F′) tg(fli1:EGFP) zebrafish embryos. Black scale bar: 100 µm. (JPG) [file pone.0058311.s001.jpg]

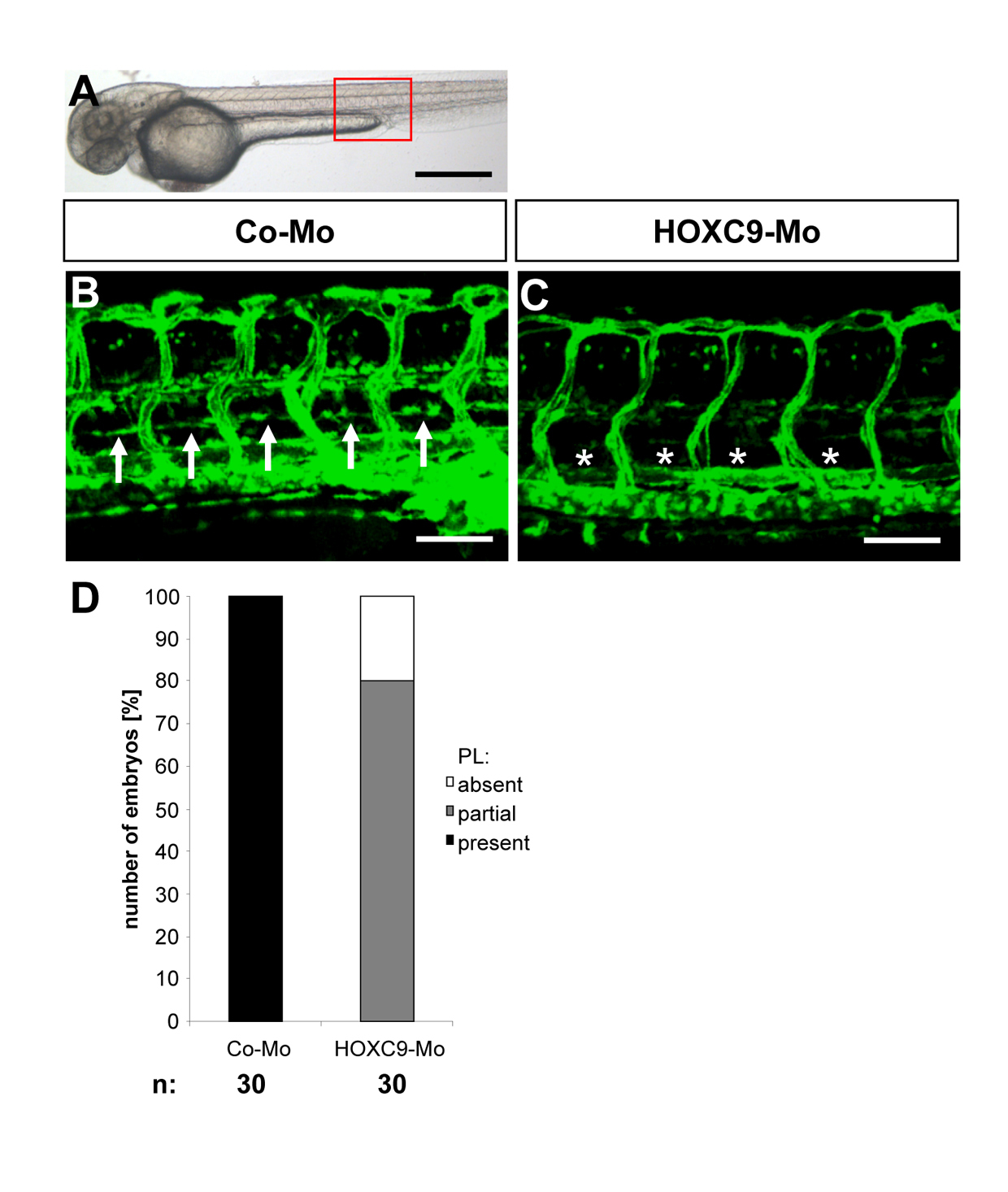

Supplement: Figure S2 — Silencing of HOXC9 expression in zebrafish inhibits assembly of parachordal lymphangioplasts (PLs) at 72 hpf. (A) Overall morphology of 72 hpf zebrafish embryo after control morpholino injection. Red box shows region displayed in (B) and (C). (B) Normal formation of the PLs (arrows) in 72 hpf tg(fli1:EGFP) zebrafish embryo after injection of 4 ng control morpholino. (C) Silencing of HOXC9 expression using 2 ng translational-blocking morpholino disrupted the formation of the PLs (asterisks) in 72 hpf tg(fli1:EGFP) zebrafish embryo. (D) Quantification of 72 hpf tg(fli1:EGFP) zebrafish embryos showing a disturbed PL formation. Embryos were divided in three groups depending on the PL appearance being completely absent, partially formed or completely present. Black scale bar: 500 µm. White scale bar: 50 µm. (JPG) [file pone.0058311.s002.jpg]

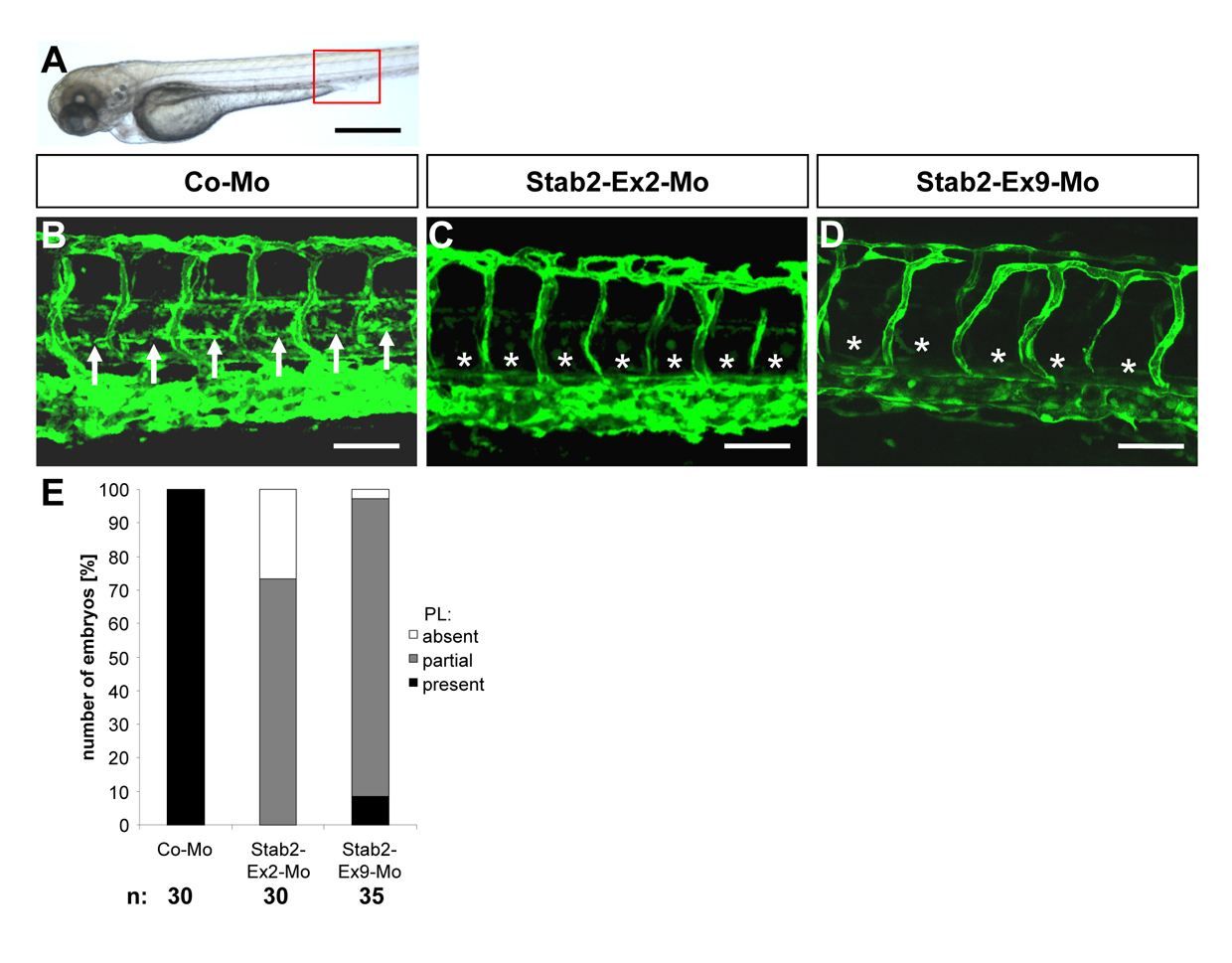

Supplement: Figure S3 — Silencing of Stab2 expression in zebrafish inhibits assembly of parachordal lymphangioplasts (PLs) at 72 hpf. (A) Overall morphology of 72 hpf zebrafish embryo after control morpholino injection. Red box shows region displayed in (B–D). (B) Normal formation of the PLs (arrows) in 72 hpf tg(fli1:EGFP) zebrafish embryo after injection of 4 ng control morpholino. (C,D) Silencing of Stab2 expression using 4 ng splice-blocking morpholino targeting exon 2 (C) or 2 ng splice-blocking morpholino targeting exon 9 (D) disrupted the formation of the PLs (asterisks) in 72 hpf tg(fli1:EGFP) zebrafish embryos. (E) Quantification of 72 hpf tg(fli1:EGFP) zebrafish embryos showing a disturbed PL formation. Embryos were divided in three groups depending on the PL appearance being completely absent, partially formed or completely present. Black scale bar: 500 µm. White scale bar: 50 µm. (JPG) [file pone.0058311.s003.jpg]

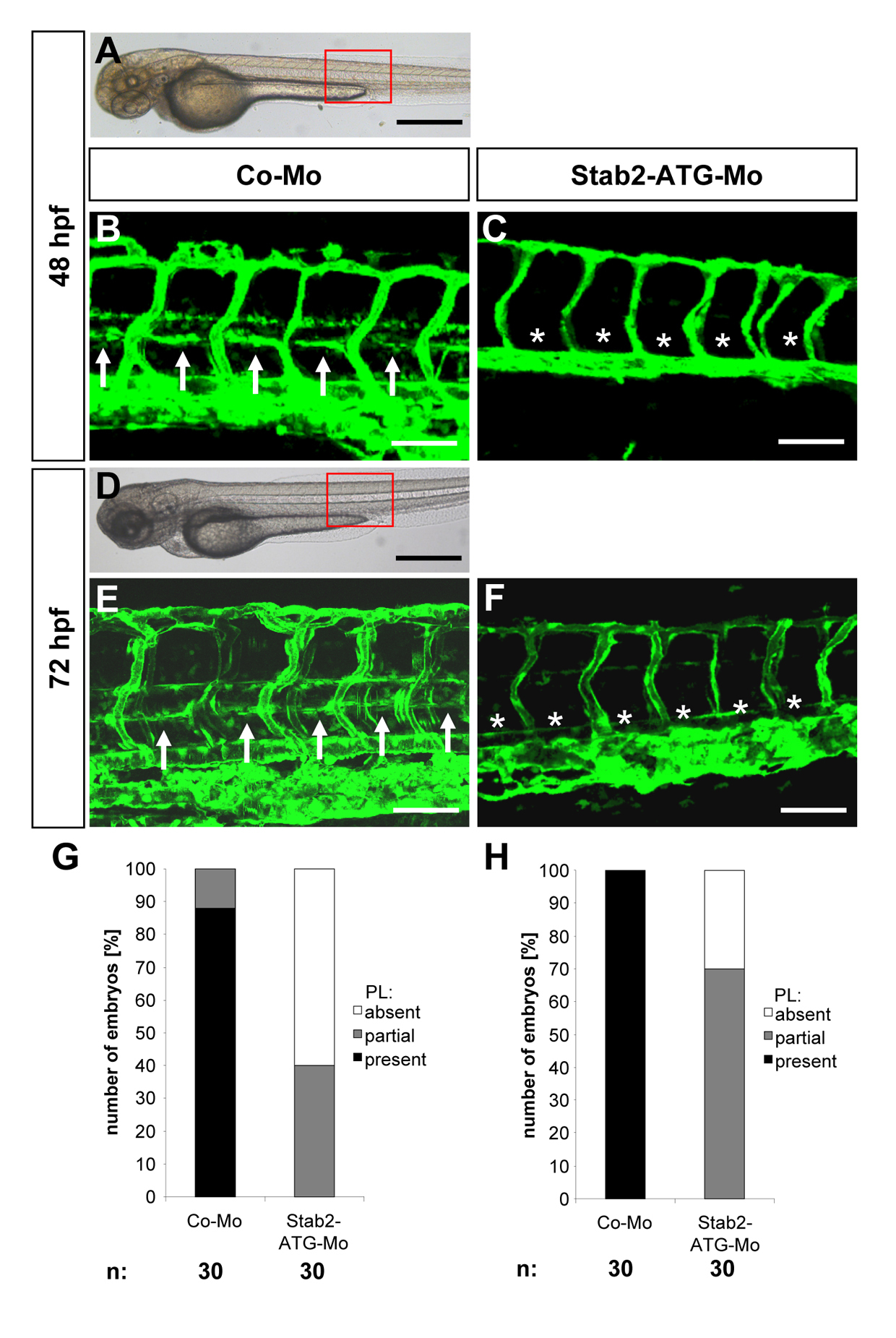

Supplement: Figure S4 — Silencing of Stab2 expression using an ATG-morpholino in zebrafish inhibits assembly of parachordal lymphangioplasts (PLs). (A;D) Overall morphology of 48 hpf (A) and 72 hpf (D) zebrafish embryos after injection of control morpholino. Red box shows region displayed below in (B,C,E,F). (B,E) Normal formation of the PLs (arrows) in 48 hpf (B) and 72 hpf (E) tg(fli1:EGFP) zebrafish embryos after injection of 4 ng control morpholino. (C,F) Silencing of Stab2 expression using 2 ng translational-blocking morpholino disrupted the formation of the PLs (asterisks) in 48 hpf (C) and 72 hpf (F) tg(fli1:EGFP) zebrafish embryos. (G,H) Quantification of 48 hpf (G) and 72 hpf (H) tg(fli1:EGFP) zebrafish embryos showing a disturbed PL formation. Embryos were divided in three groups depending on the PL appearance being completely absent, partially formed or completely present. Black scale bar: 500 µm. White scale bar: 50 µm. (JPG) [file pone.0058311.s004.jpg]

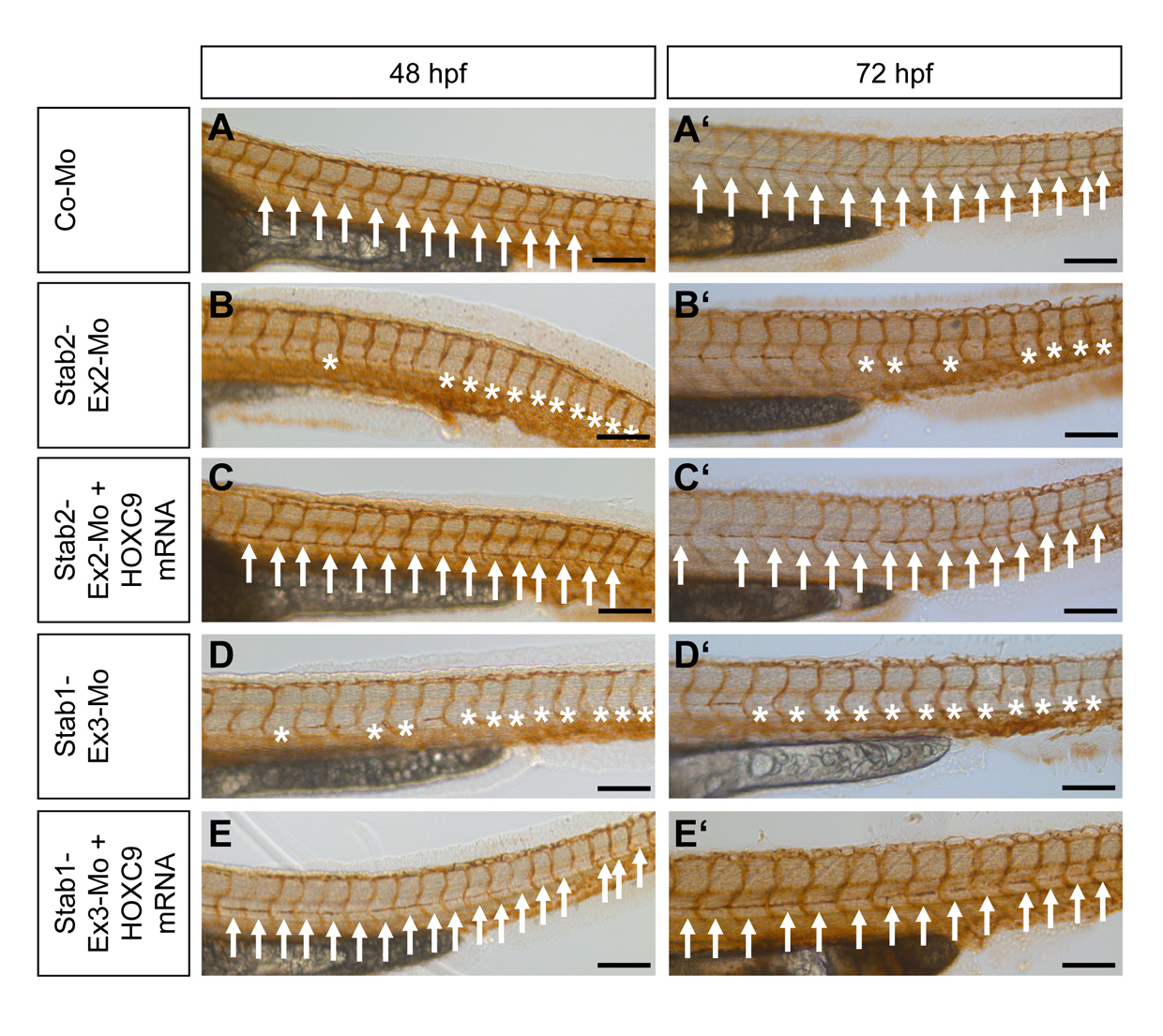

Supplement: Figure S5 — HOXC9 overexpression rescues defects in parachordal lymphangioplast (PL) formation in Stab2 and Stab1 morphants. (A–E′) Whole mount antibody staining against GFP in 48 hpf (A–E) and 72 hpf (A′–E′) tg(fli1:EGFP) zebrafish embryos injected with the indicated morpholinos. (A,A′) Normal formation of the PLs (arrows) in 48 hpf (A) and 72 hpf (A′) tg(fli1:EGFP) zebrafish embryos after injection of 4 ng control morpholino. (B,B′) Silencing of Stab2 expression using 4 ng Stab2-Ex2-Mo disrupted formation of the PLs (asterisks) in 48 hpf (B) and 72 hpf (B′) tg(fli1:EGFP) zebrafish embryos. (C,C′) Injection of HOXC9 mRNA (50 pg) rescued the Stab2 loss-of-function phenotype in 48 hpf (C) and 72 hpf (C′) tg(fli1:EGFP) zebrafish embryos. (D,D′) Silencing of Stab1 expression using 4 ng Stab1-Ex3-Mo disrupted formation of the PLs (asterisks) in 48 hpf (D) and 72 hpf (D′) tg(fli1:EGFP) zebrafish embryos. (E,E′) Injection of HOXC9 mRNA (50 pg) rescued the Stab1 loss-of-function phenotype in 48 hpf (E) and 72 hpf (E′) tg(fli1:EGFP) zebrafish embryos. Black scale bar: 500 µm. (JPG) [file pone.0058311.s005.jpg]

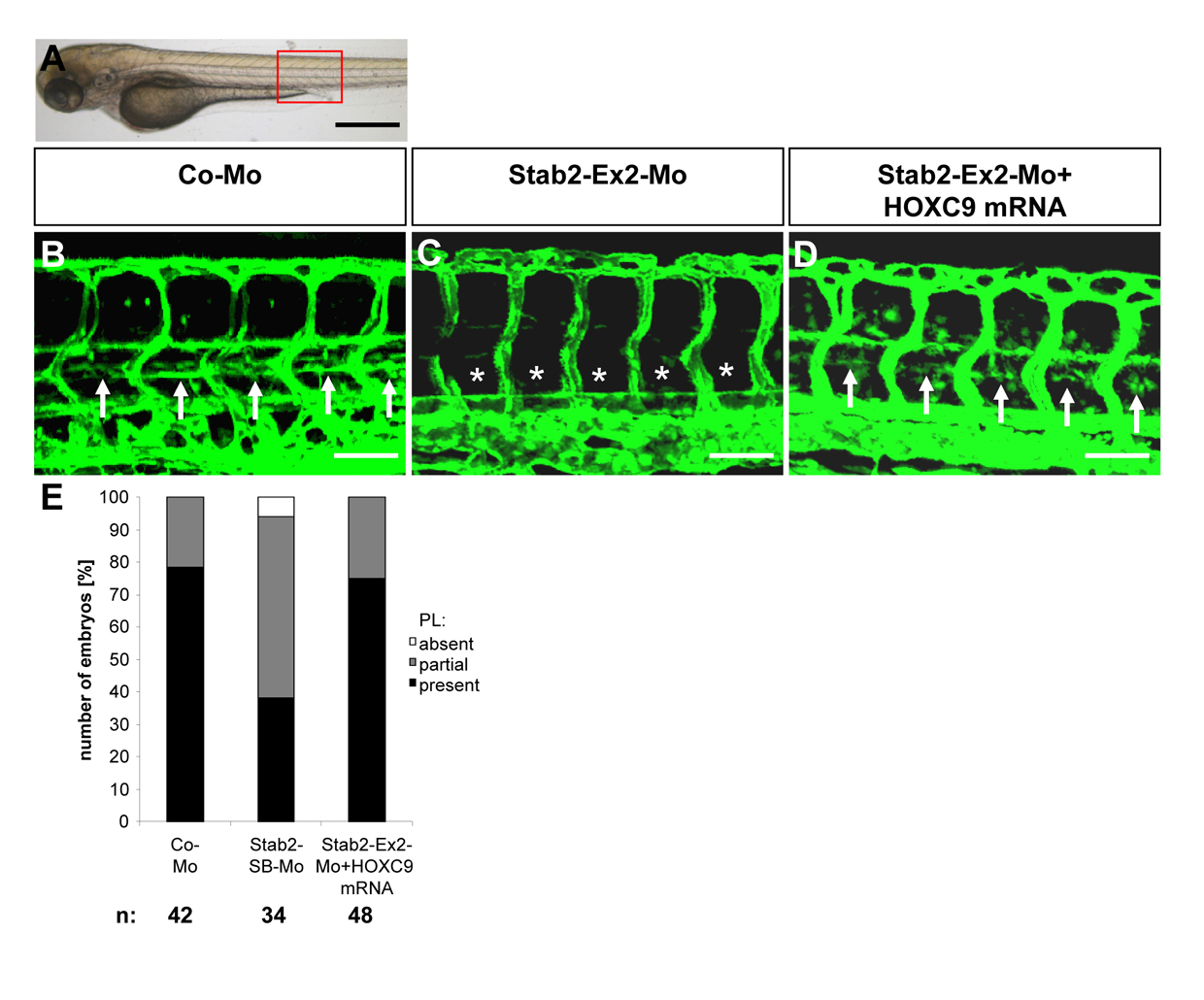

Supplement: Figure S6 — HOXC9 overexpression rescues the defects in parachordal lymphangioplast (PL) formation in Stab2 morphants at 72 hpf. (A) Overall morphology of 72 hpf zebrafish embryo after control morpholino injection. Red box shows region displayed in (B–D). (B) Normal formation of the PLs (arrows) in 72 hpf tg(fli1:EGFP) zebrafish embryo after injection of 4 ng control morpholino. (C) Silencing of Stab2 expression using 4 ng splice-blocking morpholino disrupted the formation of the PLs (asterisks) in 72 hpf tg(fli1:EGFP) zebrafish embryo. (D) Injection of HOXC9 mRNA (50 pg) rescued the Stab2 loss-of-function phenotype in 72 hpf tg(fli1:EGFP) zebrafish embryo. (E) Quantification of 72 hpf tg(fli1:EGFP) fish embryos showing a disturbed PL formation including rescue experiments using HOXC9 mRNA (50 pg). Embryos were divided in three groups depending on the PL appearance being completely absent, partially formed or completely present. Black scale bar: 500 µm. White scale bar: 50 µm. (JPG) [file pone.0058311.s006.jpg]

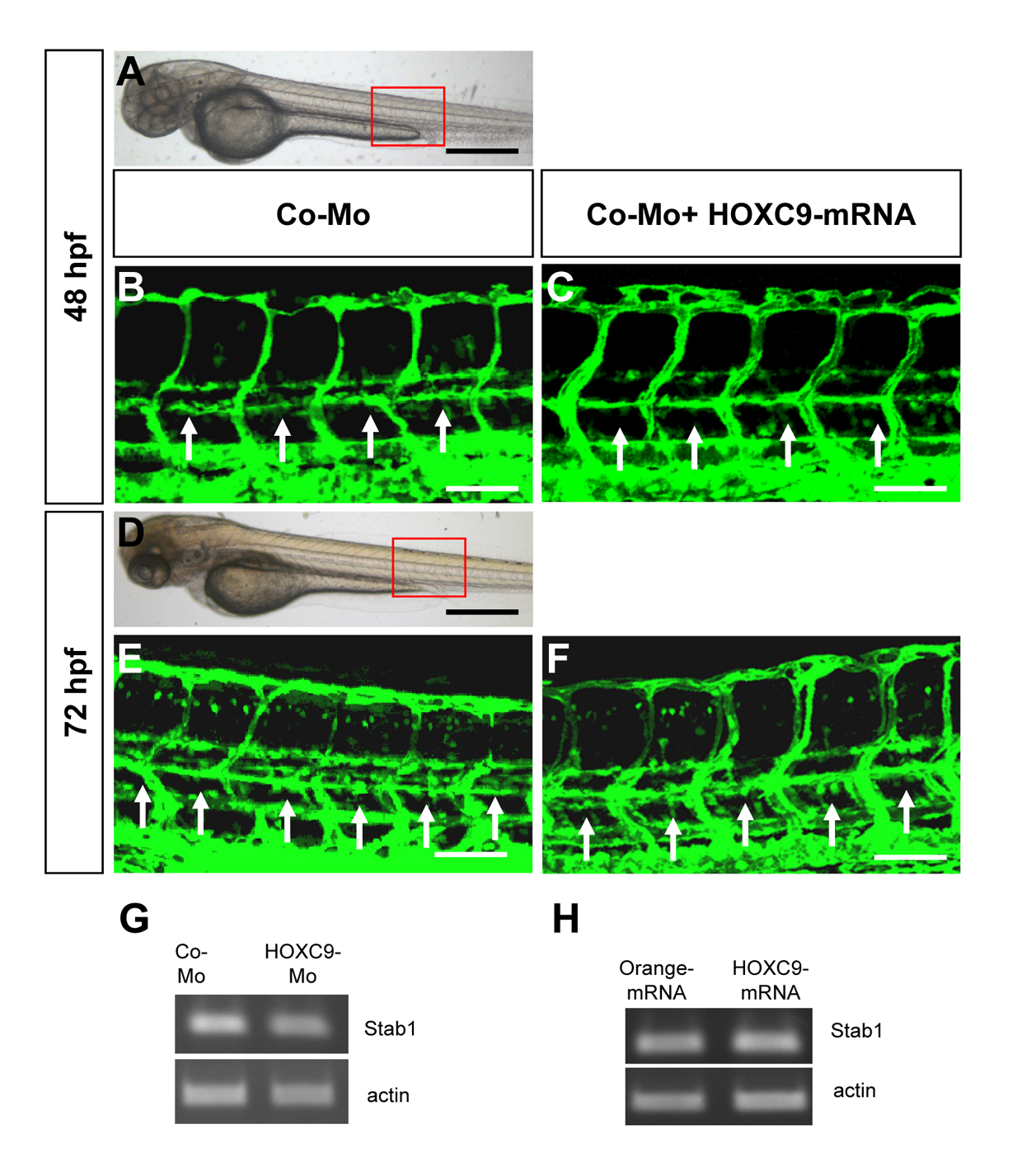

Supplement: Figure S7 — Low dose injection (50 pg) of HOXC9 mRNA shows no effect on zebrafish vascular morphology. (A,D) Overall morphology of 48 hpf (A) and 72 hpf (D) zebrafish embryos after injection of control morpholino. Red box shows region displayed in (B,C,E,F). (B,E) Normal formation of the PLs (arrows) in 48 hpf (B) and 72 hpf (E) tg(fli1:EGFP) fish embryos after injection of 4 ng control morpholino or 4 ng control morpholino combined with 50 pg HOXC9 mRNA (C,F). (G) RT-PCR analysis of zebrafish lysates injected with indicated morpholinos showed no regulation of stabilin 1 by HOXC9 silencing. (H) RT-PCR analysis of zebrafish lysates injected with indicated mRNA showed no regulation of stabilin 1 by HOXC9 overexpression. Black scale bar: 500 µm. White scale bar: 50 µm. (JPG) [file pone.0058311.s007.jpg]

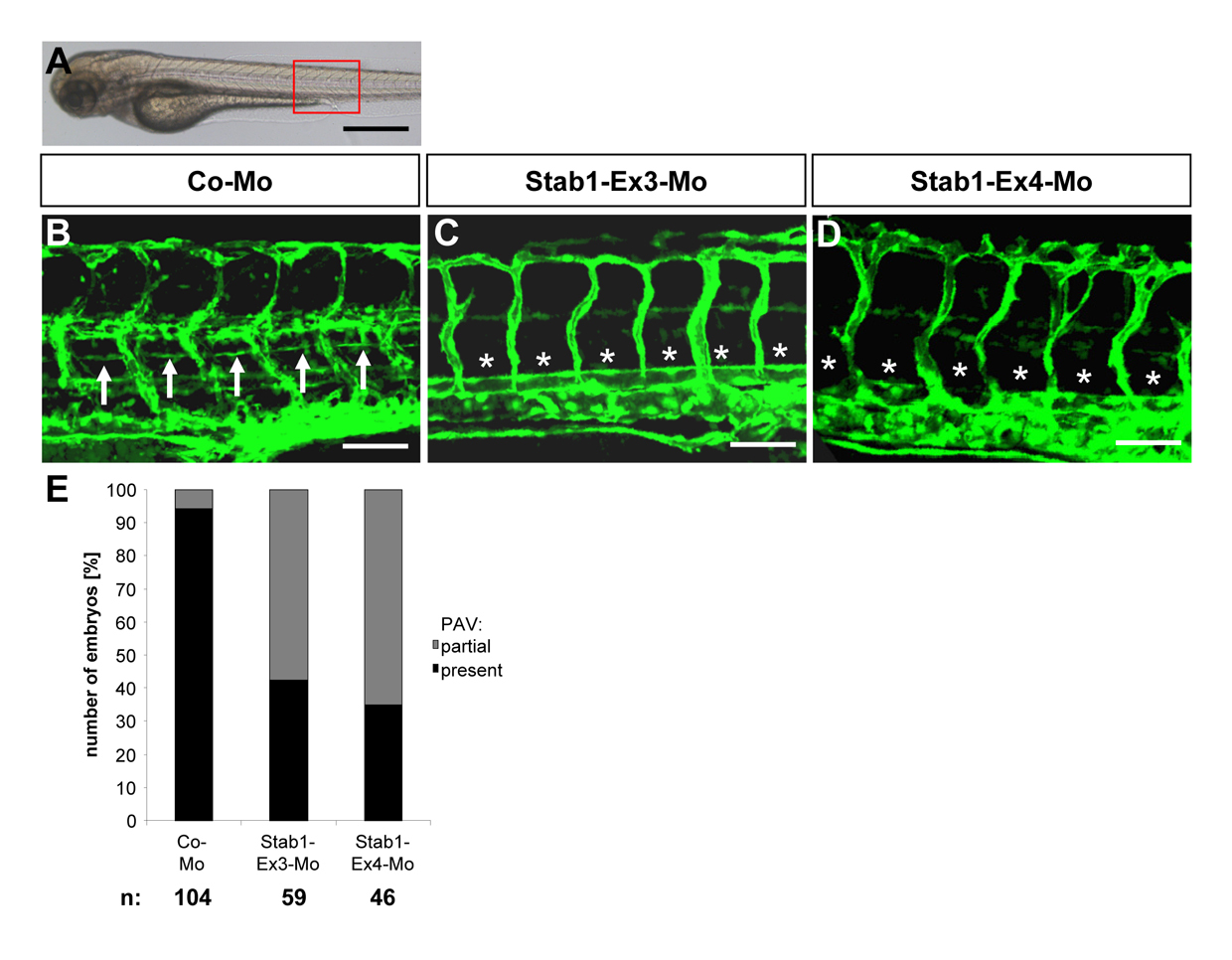

Supplement: Figure S8 — Silencing of Stab1 expression in zebrafish inhibits assembly of parachordal lymphangioplasts (PLs) at 72 hpf. (A) Overall morphology of 72 hpf zebrafish embryo after control morpholino injection. Red box shows region displayed in (B–D). (B) Normal formation of the PLs (arrows) in 72 hpf tg(fli1:EGFP) zebrafish embryo after injection of 4 ng control morpholino. (C,D) Silencing of Stab1 expression using 4 ng splice-blocking morpholino targeting exon 3 (C) or 12 ng splice-blocking morpholino targeting exon 4 (D) disrupted the formation of the PLs (asterisks) in 72 hpf tg(fli1:EGFP) fish embryos. (E) Quantification of 72 hpf tg(fli1:EGFP) fish embryos showing a disturbed PL formation. Embryos were divided in three groups depending on the PL appearance being completely absent, partially formed or completely present. Black scale bar: 500 µm. White scale bar: 50 µm. (JPG) [file pone.0058311.s008.jpg]

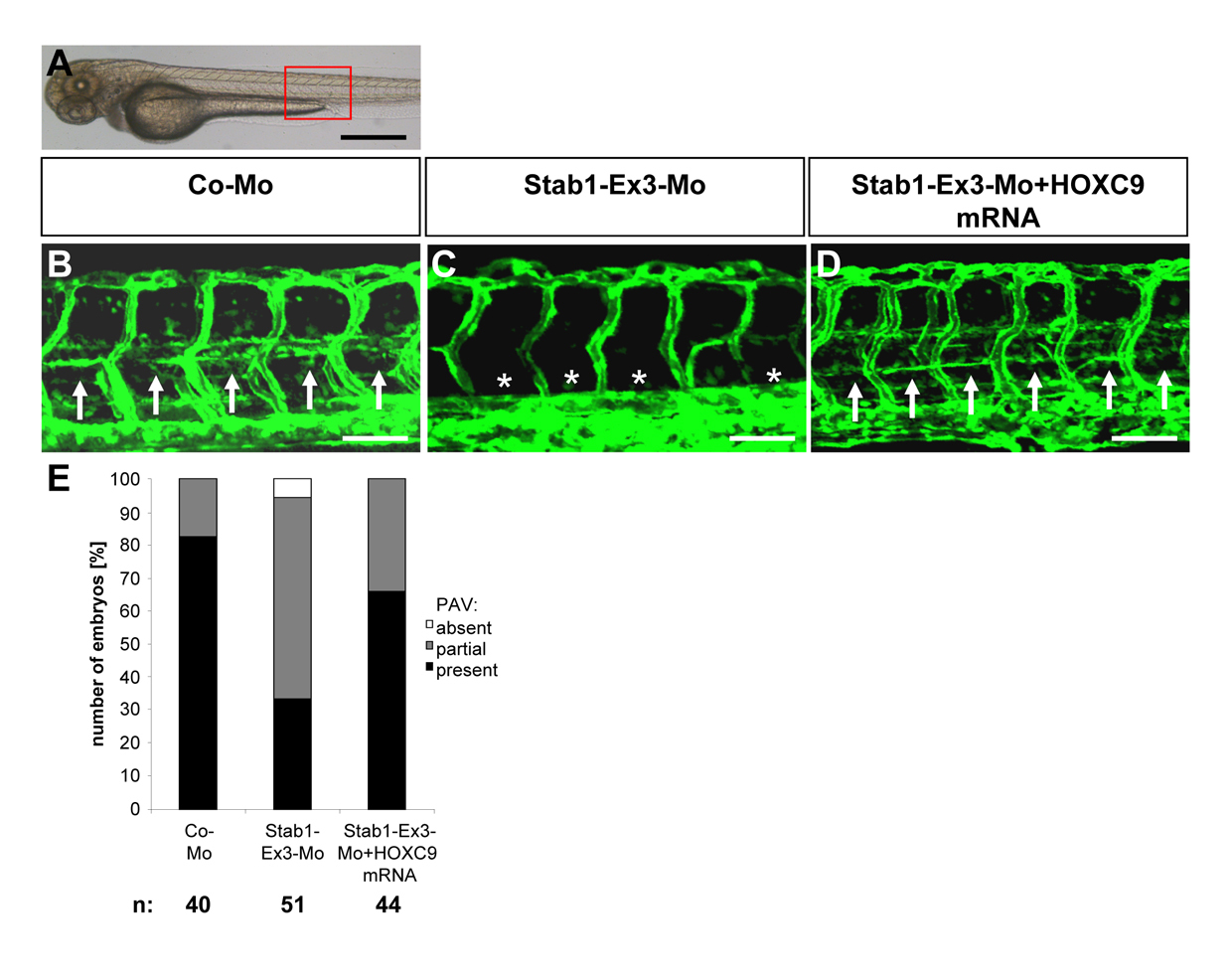

Supplement: Figure S9 — HOXC9 overexpression rescues the defects in parachordal lymphangioplast (PL) formation in Stab1 morphants. (A) Overall morphology of 72 hpf zebrafish embryo after control morpholino injection. Red box shows region displayed in (B–D). (B) Normal formation of the PLs (arrows) in 72 hpf tg(fli1:EGFP) fish embryo after injection of 4 ng control morpholino. (C) Silencing of Stab1 expression using 4 ng splice-blocking morpholino disrupted the formation of the PLs (asterisks) in 72 hpf tg(fli1:EGFP) zebrafish embryo. (D) Injection of HOXC9 mRNA (50 pg) rescued the Stab1 loss-of-function phenotype in 72 hpf tg(fli1:EGFP) zebrafish embryo. (E) Quantification of 72 hpf tg(fli1:EGFP) zebrafish embryos showing a disturbed PL formation including rescue experiments using HOXC9 mRNA (50 pg). Embryos were divided in three groups depending on the PL appearance being completely absent, partially formed or completely present. Black scale bar: 500 µm. White scale bar: 50 µm. (JPG) [file pone.0058311.s009.jpg]

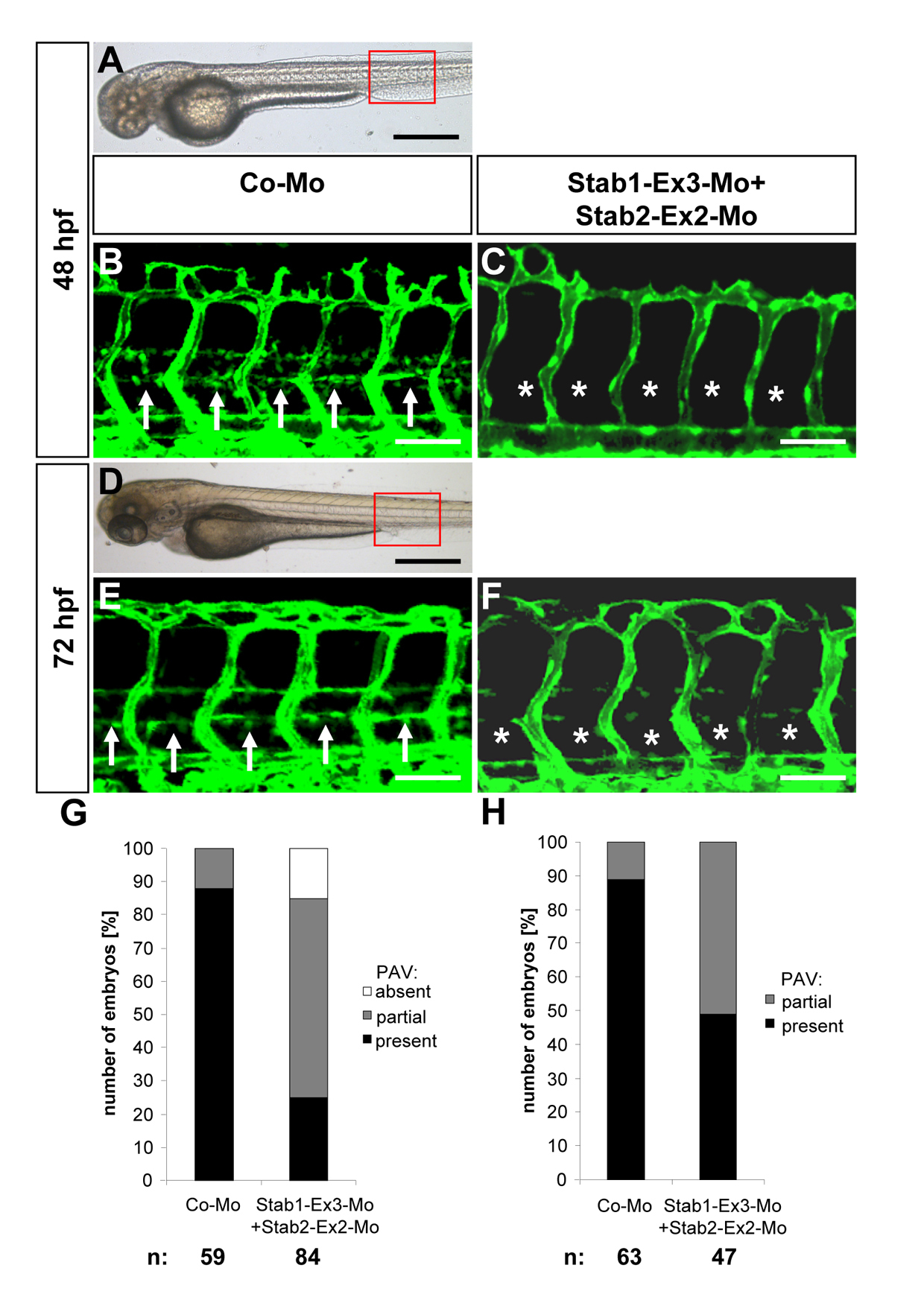

Supplement: Figure S10 — Expression silencing of Stab1 and Stab2 shows no additive effect on parachordal lymphangioplast (PL) assembly. (A,D) Overall morphology of 48 hpf (A) and 72 hpf (D) zebrafish embryos after injection of control morpholino. Red box shows region displayed in (B,C,E,F). (B,E) Normal formation of the PLs (arrows) in 48 hpf (B) and 72 hpf (E) tg(fli1:EGFP) zebrafish embryos after injection of 4 ng control morpholino. (C,F) Double silencing of Stab1 and Stab2 expression using 4 ng of each splice-blocking morpholino for Stab1 and Stab2, respectively, disrupted formation of the PLs (asterisks) in 48 hpf (C) and 72 hpf (F) tg(fli1:EGFP) zebrafish embryos. (G,H) Quantification of 48 hpf (G) and 72 hpf (H) tg(fli1:EGFP) zebrafish embryos showing a disturbed PL formation. Embryos were divided in three groups depending on the PL appearance being completely absent, partially formed or completely present. Black scale bar: 500 µm. White scale bar: 50 µm. (JPG) [file pone.0058311.s010.jpg]

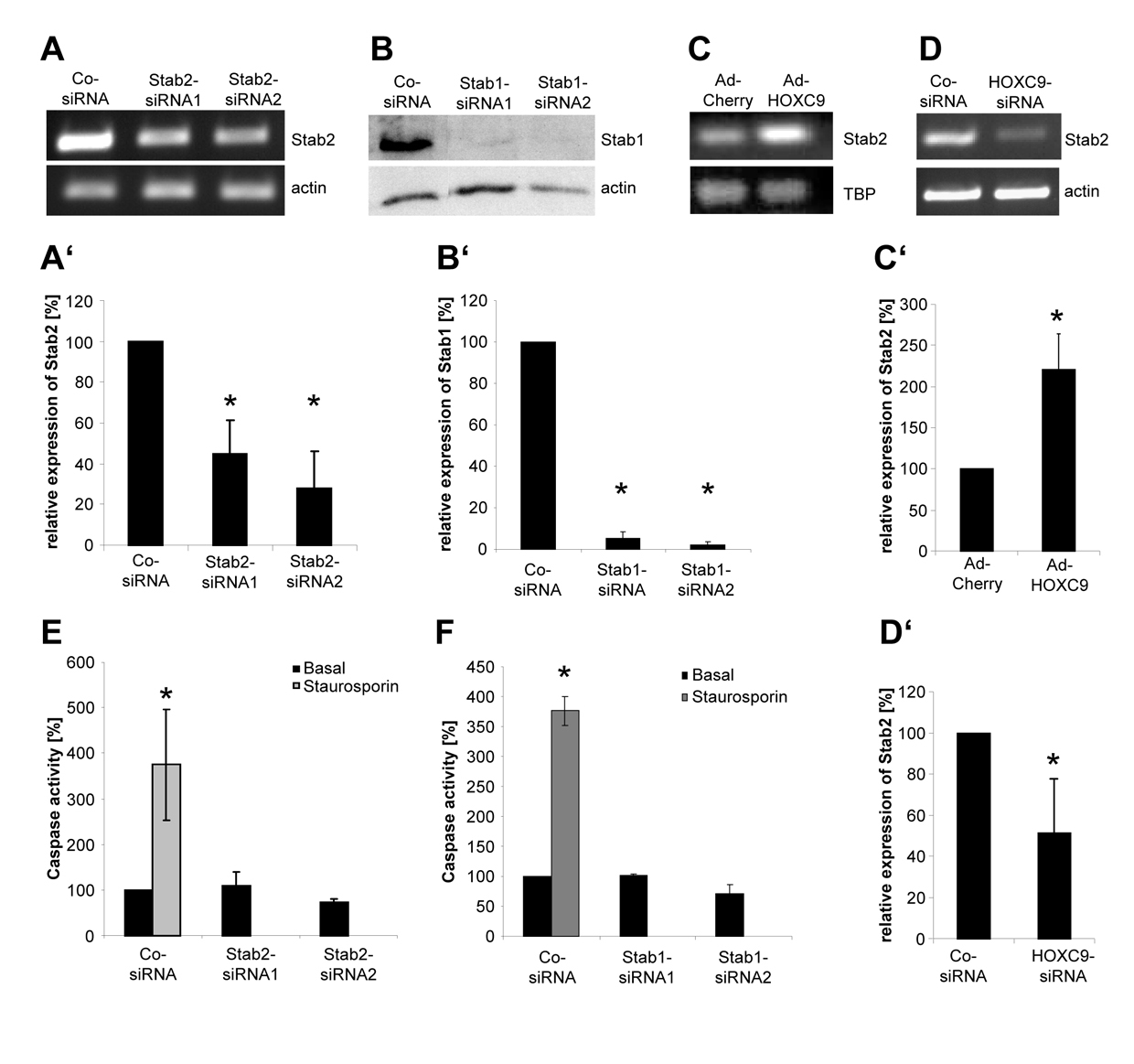

Supplement: Figure S11 — Silencing and regulation of Stab2 and Stab1 expression in endothelial cells. (A,B) Functionality of the Stab2 (A) and Stab1 (B) siRNAs. RT-PCR (A) and Western Blot (B) of HUVECs transfected with control siRNA or two different Stab2 or Stab1 siRNA. (A′,B′) Quantification of (A,B) n = 3 per group. *p<0.05 vs. control siRNA. (C) RT-PCR analysis for increased expression of Stab2 in HUVECs driven by adenovirus mediated overexpression of HOXC9. (C′) Quantification of (C), n = 3 per group. *p<0.05 vs. Ad-Cherry. (D) RT-PCR analysis for reduced expression of Stab2 in HUVECs driven by siRNA mediated silencing of HOXC9. (D′) Quantification of (D), n = 3 per group. *p<0.05 vs. Co-siRNA. (E,F) Stab2 (E) or Stab1 (F) siRNAs did not induce apoptosis in HUVECs as measured by caspase 3/7 activity. As a positive control, apoptosis in control siRNA transfected HUVECs was induced by staurosporin (n = 3 per group, *p<0.05 vs. control siRNA). (JPG) [file pone.0058311.s011.jpg]

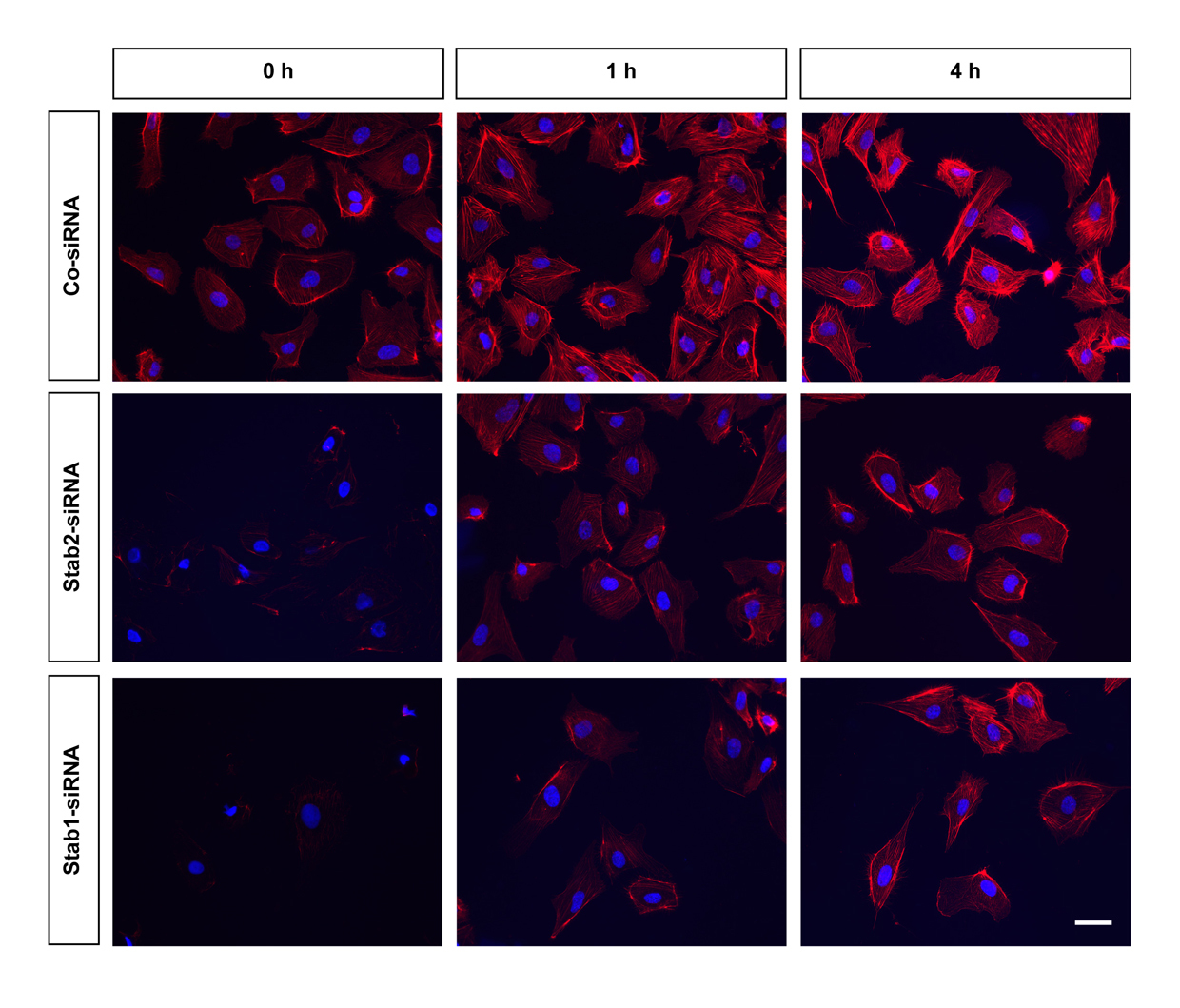

Supplement: Figure S12 — Basal and VEGF-A induced actin filament synthesis in HUVECs. HUVECs were transfected with control-, stab2- and stab1-siRNA. 48 hours after transfection, HUVECs were stimulated with VEGF-A for the indicated time points and cells were subsequently stained with phalloidin-Alexa-546. All images were captured for 100 ms. Scale bar: 100 µm. (JPG) [file pone.0058311.s012.jpg]

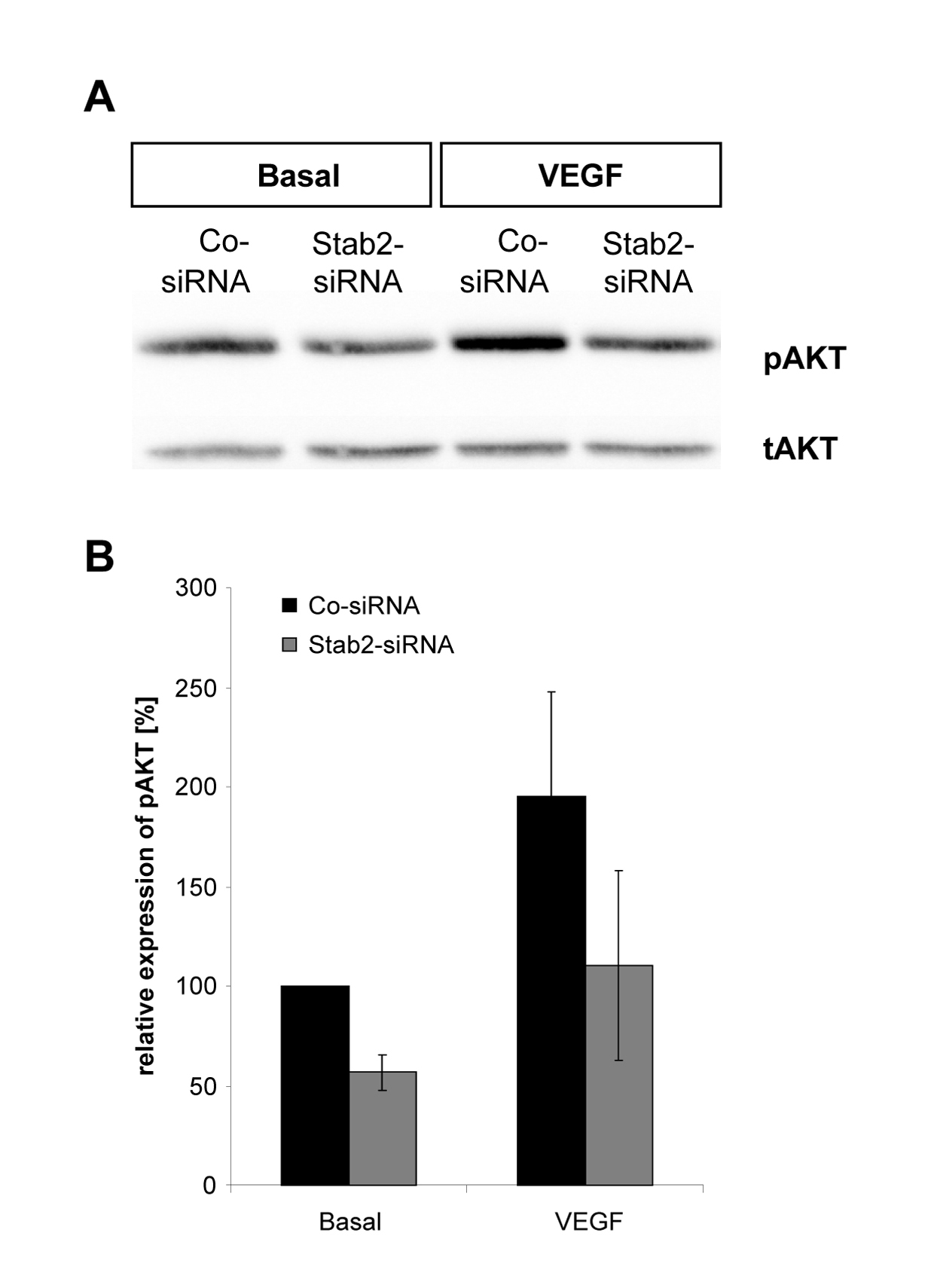

Supplement: Figure S13 — Silencing of Stab2 leads to decreased basal and VEGF-driven Akt phosphorylation in HUVECs. (A) Western blot analysis showing phosphorylated Akt (pAkt) and total Akt (tAkt). HUVECs were transfected with control- or Stab2-siRNA and subsequently stimulated with VEGF. (B) Quantification of (A), n = 3 per group. (JPG) [file pone.0058311.s013.jpg]

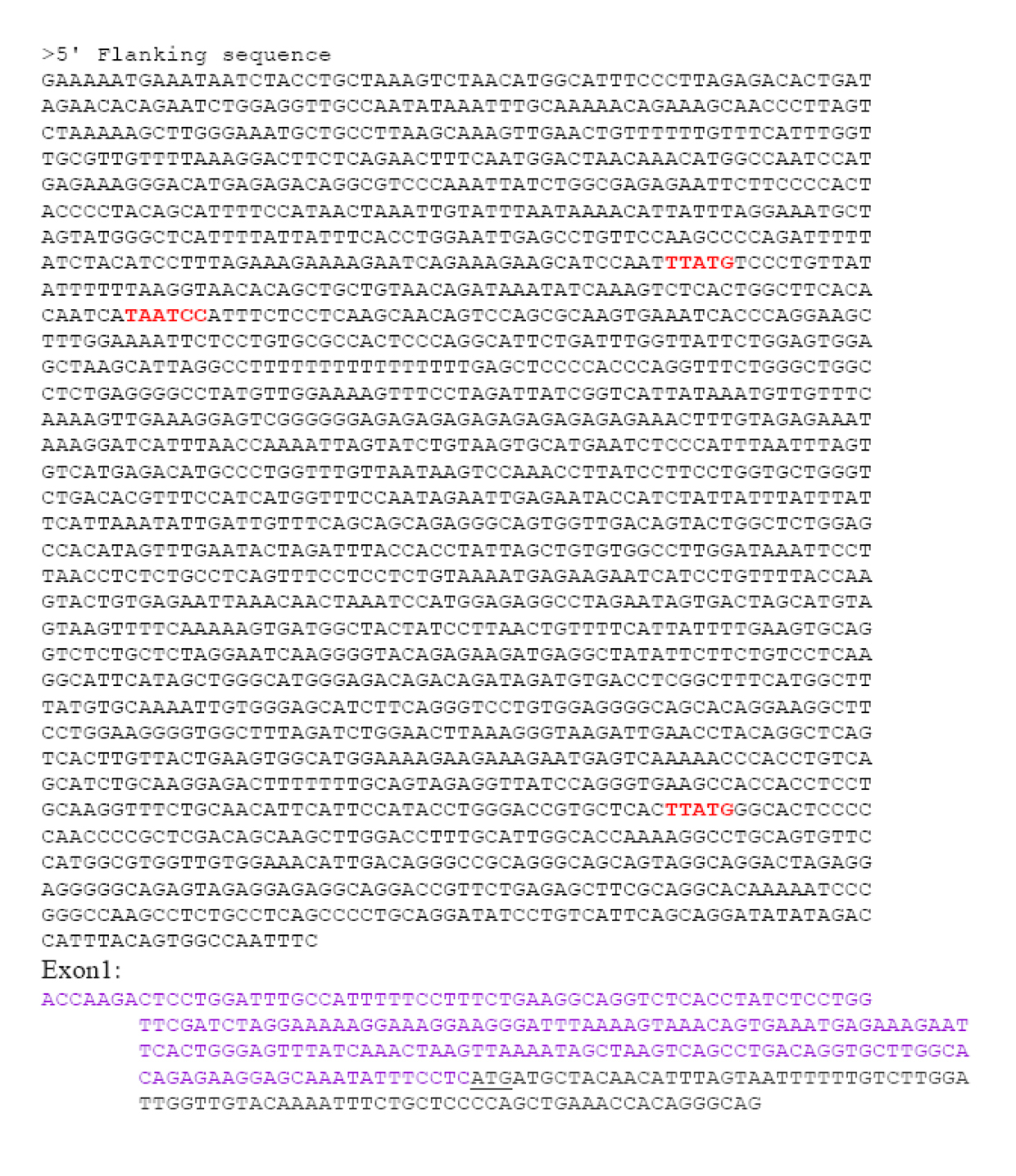

Supplement: Figure S14 — Upstream region of stabilin 2 gene with putative HOX binding sites. The region 2 kb upstream of exon 1 is displayed. The untranslated region upstream of ATG (underlined) is marked in violet. Putative HOX binding sites are highlighted in red. (JPG) [file pone.0058311.s014.jpg]
